# Supplementary material for: Real-time monitoring and feedback to improve shared decision-making for surgery (the ALPACA Study): protocol for a mixed-methods study to inform co-development of an inclusive intervention
Source: BMJ Open. 2024 Jan 18;14(1):e079155. doi: 10.1136/bmjopen-2023-079155 (PMC10806516; doi:10.1136/bmjopen-2023-079155)
Supplement: Supplementary data [file bmjopen-2023-079155supp001.pdf]

Supplemental File 1: The ALPACA Study

1. Instruments to measure the process of shared decision making

Figure S1 CollaboRATE measure

collaboRATE™

1. How much effort was made to help you understand your health issues?

0 No effort was made	1	2	3	4	5	6	7	8	9 Every effort was made

2. How much effort was made to listen to the things that matter most to you about your health issues?

0 No effort was made	1	2	3	4	5	6	7	8	9 Every effort was made

3. How much effort was made to include what matters most to you in choosing what to do next?

0 No effort was made	1	2	3	4	5	6	7	8	9 Every effort was made

From: Elwyn, Glyn, Paul James Barr, Stuart W. Grande, Rachel Thompson, Thom Walsh, and Elissa M. Ozanne. "Developing CollaboRATE: a fast and frugal patient-reported measure of shared decision making in clinical encounters." *Patient education and counseling* 93, no. 1 (2013): 102-107.

Figure S2 The 9-item Shared Decision Making Questionnaire (SDM-Q-9)

The 9-item Shared Decision Making Questionnaire (SDM-Q-9)

***[Example]* Please indicate which health complaint/problem/illness the consultation was about:**

☐

***[Example]* Please indicate which decision was made:**

☐

Nine statements related to the decision-making in your consultation are listed below. For each statement please indicate how much you agree or disagree.

		completely disagree	strongly disagree	somewhat disagree	somewhat agree	strongly agree	completely agree
**1.**	**My doctor made clear that a decision needs to be made.**	☐	☐	☐	☐	☐	☐
**2.**	**My doctor wanted to know exactly how I want to be involved in making the decision.**	☐	☐	☐	☐	☐	☐
**3.**	**My doctor told me that there are different options for treating my medical condition.**	☐	☐	☐	☐	☐	☐
**4.**	**My doctor precisely explained the advantages and disadvantages of the treatment options.**	☐	☐	☐	☐	☐	☐
**5.**	**My doctor helped me understand all the information.**	☐	☐	☐	☐	☐	☐
**6.**	**My doctor asked me which treatment option I prefer.**	☐	☐	☐	☐	☐	☐
**7.**	**My doctor and I thoroughly weighed the different treatment options.**	☐	☐	☐	☐	☐	☐
**8.**	**My doctor and I selected a treatment option together.**	☐	☐	☐	☐	☐	☐
**9.**	**My doctor and I reached an agreement on how to proceed.**	☐	☐	☐	☐	☐	☐

From: Kriston, L., Scholl, I., Hölzel, L., Simon, D., Loh, A., & Härter, M. (2010). The 9-item Shared Decision Making Questionnaire (SDM-Q-9). Development and psychometric properties in a primary care sample. *Patient education and counseling*, *80*(1), 94-99.

Figure S3 SHARED measure

SHARED – A Patient Experience of Shared Decision Making Questionnaire

What was your experience of making this treatment decision with your [health professional/s] – *for each statement, tick the box that matches best how much you agree with it:*

		Disagree Strongly	Disagree	Not Sure	Agree	Agree Strongly
	The [health] professional…					
**1**	talked about other options from the one I chose	☐	☐	☐	☐	☐
**2**	talked about whether or not there was a medically best option for my *[health problem]*	☐	☐	☐	☐	☐
**3**	gave their views about the options	☐	☐	☐	☐	☐
**4**	asked for my views about the options	☐	☐	☐	☐	☐
	I talked about…					
**5**	what was important to me about this decision	☐	☐	☐	☐	☐
**6**	why one option suited me better than another	☐	☐	☐	☐	☐
**7**	the risks and benefits of the options for me and my health	☐	☐	☐	☐	☐
	I felt…					
**8**	it would be OK to choose any option we talked about	☐	☐	☐	☐	☐
**9**	the decision made was the best one for me	☐	☐	☐	☐	☐
**10**	the *[health]* professional and I agreed which option was the best one for me	☐	☐	☐	☐	☐

From: Bekker HL, Légaré F, Nye A, Walker W. SHARED – A Patient Experience of Shared Decision Making Questionnaire. (2012). University of Leeds, UK

2. Example topic guides for qualitative data collection

Table S1 Usability testing (1.2) – interview guide

Intro

- Thank you and introduction
- Explanation of project (assess to what extent), ask if there are questions
- Ask for preferred mode for completing the survey (sms/email) and send the link
- Check if ok to audio record → *switch on recording*
- Explain purpose of the session:
  - We would like to test the survey that patients receive when booked in for surgery.
  - The focus is on **functionality**. It helps us make improvements to the process.
  - This session is **NOT** about the wording of questions, we are just interested in the usability
  - The text/email is a tester only, so the responses you give are not real
- Explain specific tasks:
  - There are two surveys. 8 steps (3 questions) for the first one, 20 steps (9 questions) for the second one.
  - We will
    - run through these steps and see how you get on with these
    - might feel a little unnatural but is important you tell me what you think and what you see, what is clear/unclear, what is easy/not straight forward or difficult to complete
  - Say where there is a problem, e.g. that you had to press twice to proceed
- Explain there will be questions at the end

Think-aloud exercise

Start with 8 steps of CollaboRATE

- Prompts if participant doesn't talk
  - Can you tell me what you currently see?
  - What are you going to do next?
  - What can you see now?
- Prompts to elicit views
  - Could you tell me what you think about Step X?
  - How do you feel about Step X?
  - What do you think about Step X?
  - How clear is Step X?
  - How easy is Step X?

Pause and ask follow-up questions

- "Having just completed the survey…"
  - How easy do you think is it to respond to the survey?
  - What do you think about the length of the survey?
  - What are your thoughts on the overall visual display? How visually appealing is the survey?
  - What would stop people from doing the survey? *Why?*
  - What issues can you think of people might encounter when completing the survey? *Why?*
  - What else would you change about how the survey is delivered? *Why?*

Table S2 Exploring views of under-served groups (1.3) – interview guide

Background

- Intro to interviewer (name, role and inability to answer care-related questions)
- Explain what SDM is and the main aim of the research project (focus on what SDM is vs isn't, current problem and relevance/importance to community)
- Reminder of anonymity, confidentiality and that interview can be stopped at any time.
- *Take questions*
- Reminder of recording and check participant is happy
- *Switch on recorder*

Explore context of SDM experience

Having just explained a bit more about what the research aims to do and what good shared decision making looks like, I am interested to hear from you...

- What is your experience with decision making for any healthcare treatment?
- How much involvement in decisions about surgical treatment would you prefer?
- Do you feel that, for whatever reason, you felt that you were/would be treated unequal to others in terms of making decision?

Exploring intervention components

- What do you think about the hospital using this survey to record people's experiences of how involved they felt in the surgical decision making?
- How do you feel about personal survey responses being passed on to the clinical team and you/the patient not being anonymous?
- What do you think needs to happen next to make sure you/ patients are given a voice and SDM is improved?
- How well do you think this process would work for everyone?
