# Supplementary material for: Real-time monitoring and feedback to improve shared decision-making for surgery (the ALPACA Study): protocol for a mixed-methods study to inform co-development of an inclusive intervention
Source: BMJ Open. 2024 Jan 18;14(1):e079155. doi: 10.1136/bmjopen-2023-079155 (PMC10806516; doi:10.1136/bmjopen-2023-079155)
Supplement: Author's manuscript [file bmjopen-2023-079155.draft_revisions.pdf]

BMJ Open is committed to open peer review. As part of this commitment we make the peer review history of every article we publish publicly available.

When an article is published we post the peer reviewers' comments and the authors' responses online. We also post the versions of the paper that were used during peer review. These are the versions that the peer review comments apply to.

The versions of the paper that follow are the versions that were submitted during the peer review process. They are not the versions of record or the final published versions. They should not be cited or distributed as the published version of this manuscript.

BMJ Open is an open access journal and the full, final, typeset and author-corrected version of record of the manuscript is available on our site with no access controls, subscription charges or pay-per-view fees (http://bmjopen.bmj.com).

If you have any questions on BMJ Open's open peer review process please email info.bmjopen@bmj.com

BMJ Open

Protocol for a mixed-methods study to inform co-development of an inclusive intervention that uses real-time monitoring and feedback to improve shared decision making for surgery (the ALPACA study)

Journal:	*BMJ Open*
Manuscript ID	bmjopen-2023-079155
Article Type:	Protocol
Date Submitted by the Author:	23-Aug-2023

Complete List of Authors:	Hoffmann, Christin; NIHR Bristol Biomedical Research Centre, Bristol Centre for Surgical Research, Bristol Medical School: Population Health Science, University of Bristol<br>Avery, Kerry; NIHR Bristol Biomedical Research Centre, Bristol Centre for Surgical Research, Bristol Medical School: Population Health Sciences, University of Bristol<br>Macefield, Rhiannon; NIHR Bristol Biomedical Research Centre, Bristol Centre for Surgical Research, Bristol Medical School: Population Health Science, University of Bristol<br>Snelgrove, Val; Patient representative<br>Blazeby, Jane; NIHR Bristol Biomedical Research Centre, Bristol Centre for Surgical Research, Bristol Medical School: Population Health Science, University of Bristol<br>Hopkins, Della; North Bristol NHS Trust<br>Hickey, Shireen; Bradford Teaching Hospitals NHS Foundation Trust<br>Cabral, Christie; University of Bristol Centre for Academic Primary Care, Bristol Medical School<br>Hall, Jennifer; Bradford Teaching Hospitals NHS Foundation Trust,<br>Gibbison, Ben; University of Bristol, ;<br>Rooshenas, Leila; NIHR Bristol Biomedical Research Centre, Bristol Centre for Surgical Research, Bristol Medical School: Population Health Science, University of Bristol<br>Williams, Adam; North Bristol NHS Trust Southmead Hospital<br>Aning, Jon; North Bristol NHS Trust, Bristol Urological Institute, Southmead Hospital; University of Bristol, Population Health Sciences, Bristol Medical School<br>Bekker, Hilary; University of Leeds, Leeds Unit of Complex Intervention Development (LUCID), Leeds Institute of Health Sciences, School of Medicine; Aarhus Universitet, The Research Centre for Patient Involvement (ResCenPI), Department of Public Health<br>McNair, Angus; University of Bristol, Centre for Surgical Research, Bristol Medical School: Population Health Sciences; North Bristol NHS Trust, GI Surgery<br>Judge, Andrew; University of Bristol, Musculoskeletal Research Unit<br>Smith, Andrew; North Bristol NHS Trust<br>Lingampalli, Archana; North Bristol NHS Trust<br>Reeves, Barnaby; University of Bristol, Bristol Trials Centre, Population Health Sciences, Bristol Medical School

Preshaw, Jessica; North Bristol NHS Trust
Whitehouse, Michael; University of Bristol, Musculoskeletal Research Unit, Translational Health Sciences, Bristol Medical School; North Bristol NHS Trust
Cresswell, Paul; North Bristol NHS Trust
Braude, Philip; North Bristol NHS Trust
Potter, Shelley; University of Bristol, Translational Health Sciences, Bristol Medical School; North Bristol NHS Trust
Beckitt, Timothy; North Bristol NHS Trust
Whittlestone, Timothy; North Bristol NHS Trust

Keywords: SURGERY, Decision Making, Patient Participation, Patient Reported Outcome Measures

SCHOLARONE™ Manuscripts

Protocol for a mixed-methods study to inform co-development of an inclusive intervention that uses real-time monitoring and feedback to improve shared decision making for surgery (the ALPACA study).

Submission category: Protocol

Authors:

Christin Hoffmann[1], Kerry Avery[1], Rhiannon Macefield[1], Val Snelgrove[2], Jane Blazeby[1], Della Hopkins[3], Shireen Hickey[4], Christie Cabral[5], Jennifer Hall[4], Ben Gibbison[1,6], Leila Rooshenas[1], Adam Williams[3], Jonathan Aning[7, 8], Hilary L Bekker[9, 10], Angus GK McNair[1,3] on behalf of the ALPACA study team:

Andy Judge[1];
Andrew Smith[3];
Archana Lingampalli[3];
Barnaby Reeves[11];
Jessica Preshaw[3];
Michael R Whitehouse[1,3];
Paul Cresswell[3];
Philip Braude[3];
Shelley Potter[3];
Timothy Beckitt[3];
Timothy Whittlestone[3]

Author affiliations:

[1] National Institute for Health and Care Research Bristol Biomedical Research Centre, Bristol Centre for Surgical Research, Bristol Medical School: Population Health Sciences, University of Bristol, Bristol, BS8 2PS, UK

[2] Patient Representative

[3] North Bristol NHS Trust, Bristol, UK

[4] Bradford Institute for Health Research, Bradford Teaching Hospitals NHS Foundation Trust, Bradford, UK

[5] Centre for Academic Primary Care, Bristol Medical School, University of Bristol, Bristol, UK

[6] University Hospitals Bristol and Weston NHS Foundation Trust, Bristol, UK

[7] Bristol Urological Institute, Southmead Hospital, North Bristol Trust, Bristol, UK

For peer review only

[8] Population Health Sciences, Bristol Medical School, University of Bristol, Bristol, UK

[9] Leeds Unit of Complex Intervention Development (LUCID), Leeds Institute of Health Sciences, School of Medicine, University of Leeds, Leeds, UK

[10] The Research Centre for Patient Involvement (ResCenPI), Department of Public Health, Aarhus University, Central Denmark Region, DK

[11] Bristol Trials Centre, Population Health Sciences, Bristol Medical School, University of Bristol, Bristol, UK

Correspondence: Christin Hoffmann (c.hoffmann@bristol.ac.uk)

Running title: The ALPACA feasibility study protocol

Keywords: Shared decision making; Mixed-methods; Intervention development; Inequalities; Underserved groups; Outcome measurement; Protocol; Surgery; ePROMS; electronic measurement

Word count: 3,862

For peer review only

ABSTRACT

Introduction: High-quality shared decision making (SDM) is a priority of health services, but only achieved in a minority of surgical consultations. Improving SDM for surgical patients may lead to more effective care and moderate the impact of treatment consequences. There is a need to establish effective ways to achieve sustained and large-scale improvements in SDM for all patients whatever their background. The ALPACA study aims to develop, pilot, and evaluate a decision support intervention that uses real-time feedback of patients' experience of SDM to change patients' and healthcare professionals' decision-making processes before adult elective surgery and to improve patient and health service outcomes.

Methods and analysis: This protocol outlines a mixed-method study, involving diverse stakeholders (adult patients, healthcare professionals, members of the community) and three NHS trusts in England. Detailed methods for the assessment of the feasibility, usability, and stakeholder views of implementing a novel system to monitor the SDM process for surgery automatically and in real-time are described. The study will measure the SDM process using validated instruments (CollaboRATE, SDM-Q-9, SHARED-Q10) and will conduct semi-structured interviews and focus groups to examine 1) the feasibility of automated data collection, 2) the usability of the novel system, and 3) the views of diverse stakeholders to inform the use of the system to improve SDM. Future phases of this work will complete the development and evaluation of the intervention.

Ethics and dissemination: Ethical approval was granted by the NHS HRA North West - Liverpool Central Research Ethics Committee (reference: 21/PR/0345). Approval was also granted by North Bristol NHS Trust to undertake quality improvement work (reference: Q80008) overseen by the consent & SDM programme board and reporting to an Executive Assurance Committee.

For peer review only

Strengths and limitations of this study

- The ALPACA Study will develop a novel automated system to monitor shared decision making in real-time across multiple UK NHS trusts and surgical specialties.
- A mixed-methods study design will use a diverse representative sample of surgical patients to determine the feasibility of data collection, the usability of the novel system and understand views of diverse stakeholders to inform use of the system.
- Recruitment will focus on recognised under-served groups (economically disadvantaged, older age, ethnic minority) and involvement from a range of NHS trusts, including Bristol and Bradford to maximise reach to an ethnically and socio-economically diverse population.
- The research was conceived and planned through collaboration with patient partners who were actively involved in the study design and provide continued oversight.
- The study uses three validated questionnaires to monitor SDM (CollaboRATE, SDM-Q-9, SHARED-Q10), including first use of the SHARED-Q10 measure in a surgical setting.
- This study excluded patients without capacity due to distinct requirements and guidance for consent and shared decision making processes in this population.

For peer review only

For peer review only - http://bmjopen.bmj.com/site/about/guidelines.xhtml

Introduction

Shared decision making (SDM) is a process where patients are supported to reach decisions in collaboration with health professionals [1]. Global and United Kingdom (UK) policy [2–4], professional and regulatory guidelines [5,6] recommend SDM in all healthcare settings. Making good decisions is particularly important for the five million people per year deciding to have surgery in the UK because, unlike many medical therapies, the effects are usually immediate and irreversible. Ensuring patients and surgeons have discussed accurate information about all options and their consequences, exchanged their reasoning about, and preferences for, each option, and agreed the treatment plan is essential to a good, SDM process.

Evidence shows there is scope to improve SDM for surgery. A systematic review of 22 surgical studies found that only 36% of 13,176 patients perceived their consultation as shared [7]. Other systematic reviews show that surgeons underestimate patients' information needs [8], and patients do not receive desired information before surgery [9]. Major surgical risks go undisclosed [10], and patients report feeling uninformed [11] and want more involvement in decision-making [12]. The impact of these deficiencies is inadequately understood. It is thought that improving SDM processes may lead to more effective care through enhanced clinician-patient reasoning [13], thereby supporting treatment choices with greater benefit/harm ratios [8] and reducing overall use of health services [14,15]. High-quality SDM may also moderate the impact of treatment harms through more realistic treatment expectations [16,17] and improved self-management [18].

Guidelines for the implementation of SDM have been recently published by the National Institute for Health and Care Excellence (NICE) that includes best-evidence from a Cochrane review [9] and consultation with 454 stakeholders. It concluded that a combination of interventions to support organisations, clinicians, and patients are needed, but the evidence for these interventions is often poor [19]. Key priority areas were identified for future research, including generating evidence about how to: sustain SDM implementation at an organisation/health service level; measure the effectiveness of the SDM process for different contexts/settings/people; and ensure the SDM process is inclusive of people from diverse backgrounds (e.g. ethnic minorities, persons of lower health literacy or income backgrounds).

The ALPACA Study aims to address these deficiencies. We will develop, pilot, and evaluate a decision support intervention that uses real-time feedback of patient experiences of the SDM process to impact patient and professional decision-making processes before adult elective surgery and improve patient and health service outcomes. The intervention will include 1) efficient, real-time

For peer review only - http://bmjopen.bmj.com/site/about/guidelines.xhtml

evaluation of patient experiences of SDM at scale, 2) timely feedback of individual patient-reported experiences of SDM to care teams before surgery and 3) activities supporting meaningful change in patient and professional decision-making about surgery, individually and together.

This project aims to enable surgical teams to remedy deficiencies in the SDM process before surgery and thereby addresses NICE research priorities to detect such deficits reported by the patient. The intervention will be deliverable at scale to create sustained improvement in SDM through system-wide changes in decision-making processes facilitated by continuous patient-reported feedback. It will be co-created with patients with a focus on inclusivity of recognised under-served groups. Developing methods for efficient evaluation of the SDM process will make measurement of SDM outcomes more consistent and meaningful.

Aim and objectives

The overall aim of this project is to develop, pilot, and evaluate a decision support intervention that uses real-time feedback of patient experience of SDM to change patient and professional decision-making processes before adult elective surgery and improve patient and health service outcomes. There are three phases with the following objectives:

Phase 1: Assess the feasibility, usability and stakeholder views of implementing an automated system to monitor the SDM process for surgery in real-time.

Phase 2: Co-develop and refine the intervention with patients and professionals to understand how the intervention works, for whom, and in what context using findings from Phase 1.

Phase 3: Evaluate the effectiveness, cost-effectiveness, and implementation of the intervention to improve patient and health service outcomes in the English NHS.

This protocol describes phase 1. Details of subsequent phases which will complete the development (Phase 2) and evaluation of the intervention (Phase 3) will be described in future publications.

Methods

For peer review only - http://bmjopen.bmj.com/site/about/guidelines.xhtml

For peer review only

The project will employ mixed-methods to develop a complex intervention comprising multiple components that will impact a wide range of stakeholders and system processes. The overall aim to develop and evaluate the intervention will be conducted according to Medical Research Council (MRC) guidelines [20]. Phase 1 reported here is consistent with the MRC framework's feasibility phase, with consideration of the core elements critical for complex intervention research. Any qualitative elements will be reported in accordance with the consolidated criteria for reporting qualitative studies (COREQ) guidelines [21].

Conceptualisation

There is no unified definition of SDM. A systematic review identified 40 SDM models currently available with 53 different elements clustered in 24 overarching components [1]. Components present in more than half of models were: 'describe treatment options' (88% of models); 'make a decision' (75%); 'patient preferences' (68%); 'tailor information' (65%); 'deliberate' (58%); 'create choice awareness' (55%) and 'learn about the patient' (55%).

This study will conceptualise SDM using the 'Three Talk model' (2012) [22], later refined to 'Implement-SDM' (2019) [23]. This single-component model provides a guide for enhancing health professional communication to deliver SDM, and is the most highly referenced model (>1800 citations, Web of Science). It involves three key steps consistent with other models of SDM: 1) introduction of choice, 2) describing options and 3) helping patients explore preferences and make decisions. This was the chosen model for an NHS MAGIC programme [7] and is recommended in NICE guidelines [24].

Setting

Research will be conducted at three UK hospital trusts (North Bristol Trust/NBT, University Hospitals Bristol and Weston NHS Foundation Trust/UHBWFT, and Bradford Teaching Hospitals NHS Foundation Trust/BTHFT). NBT is one of the largest acute NHS trusts in the UK [25]. It provides a full range of acute clinical care for both local and regional clinical commissioning groups in South-West England. Specialised services are provided through NHS England, Welsh Health Boards and Welsh Specialist Commissioners. Services provided include elective and emergency gastrointestinal surgery, obstetrics and gynaecology, as well as specialist regional services in urology, neurosciences, trauma and orthopaedic and vascular surgery. One UHBWFT department is included as the South-West England regional cardiac surgical centre. BTHFT is an acute Trust in the North of England with a full

For peer review only - http://bmjopen.bmj.com/site/about/guidelines.xhtml

For peer review only

range of elective and emergency surgical services. Bristol and Bradford were purposively selected to maximise reach to ensure a diverse representative sample is included (e.g. 26.8% classed as Asian or Asian British, compared with 5.5% in Bristol).

Phase 1: Assess the feasibility, usability and stakeholder views of implementing an automated system to monitor the SDM process for surgery in real-time

Phase 1 will determine whether it is feasible and acceptable to monitor SDM processes for surgery automatically and in real-time using a novel electronic system. Objectives are to explore:

1.1 Feasibility of automated data collection

1.2 Usability of the electronic measurement system

1.3 Views of diverse stakeholders to inform the use of the system to improve SDM

Each objective comprises separate methods which are described in turn below. This phase is expected to continue until June 2025.

1.1 Feasibility of automated data collection

Feasibility assessment is designed to establish the feasibility of automated real-time evaluation of patient experiences of SDM at scale and will identify opportunities to optimise recruitment and data collection.

Participants

All patients over the age of 18 who have been booked for planned vascular, gastrointestinal, urological, neurosurgical, gynaecological, breast, cardiac and orthopaedic surgical procedures at participating hospitals will be eligible to participate. Surgical departments have been selected to be broadly representative of a diverse range of surgical specialties. Excluded will be patients under the age of 18, those without capacity to consent for medical procedures, or undergoing unplanned (emergency) surgery or endoscopic procedures.

Measurement of patient experience

For peer review only - http://bmjopen.bmj.com/site/about/guidelines.xhtml

Real-time measurement of patient experiences of SDM will be facilitated by a secure, automated system procured through a commercial software provider approved by NHS trusts. Eligible patients will be identified through routine electronic patient records (EPR) using algorithms developed in collaboration with the software provider. Structured data queries will be designed to extract details of patients booked for eligible procedures. Queries will be designed to run automatically, securely transferring data from the hospital to the software provider daily to account for changes in scheduling. The automated system will send three validated SDM measurement instruments by short messaging service (SMS) or email within one day after surgery booking (baseline). A reciprocal data feed will securely return patients' survey responses to the hospital data warehouse for secure storage. Follow-up measures are sent the day before surgery by either SMS or email.

Selection of three SDM measures was made through discussions within the study team and were informed by a systematic review of SDM measurement instruments using COSMIN (consensus-based standards for the selection of health measurement instruments) methods [26], national guidelines [19] and recommendations and use within NHS clinical practice [27–29]. The CollaboRATE instrument is a validated 3-item patient-reported measure assessing the extent of SDM experienced by patients [30]. Assessment of the instrument using COSMIN methods demonstrated acceptable discriminative validity, concurrent validity, intra-rater reliability and sensitivity to change [26]. It has been used in excess of 40 studies [31]. The SDM-Q-9 instrument is a validated 9-item patient-reported measure that evaluates their perceptions of involvement in the decision-making process [32]. It has been widely used in interventional studies and demonstrates good reliability, structural validity [26,32]. The SHARED-Q10 instrument is a 10-item patient-reported measure to assess patient perceptions of information provided, involvement in consultations, and agreement with the decision made [33]. It was developed, validated, and used in the NHS Rightcare programme [34] and evaluates domains beyond patient perception of professional communication.

Analysis

Feasibility of real-time monitoring will be evaluated by analysis of overall recruitment rate and response rates to the SDM measures at baseline and follow-up. Response rates will be presented as a number and percentage based on patients who completed the measures (e.g. completed all three items of the CollaboRATE instrument). Issues of equality, diversity and inclusion will be explored by examining the correlation between responders/non-responders and sociodemographic patient variables extracted from EPR.

For peer review only

For peer review only - http://bmjopen.bmj.com/site/about/guidelines.xhtml

Relationships between responders and non-responders and clinical and sociodemographic details will be explored using uni- and multivariable logistic regression. Included will be age, sex, ethnicity, index of multiple deprivation, and clinical and treatment parameters will be explored (e.g. operation (three-digit OPCS code), diagnosis (ICD10), date of booking, specialty, number of outpatient appointments in relevant specialty, number and length of hospital inpatient episode in relevant specialty). Index of multiple deprivation will be derived by Lower Layer Super Output Area (LSOAs) for individuals' postcode. All variables will be extracted from routinely collected data in EPR.

In addition, the study team will document any relevant technical, financial, administrative, and logistical observations throughout the study and pertinent challenges using shared electronic records (e.g. Microsoft Office suite). Any learning points will be descriptively summarised.

1.2 Usability of the electronic measurement system

Usability testing will be conducted according to International Standards Organisation (ISO) standards for human-systems interaction (9241-11:2018) [35,36]. System users, defined as anyone who is a current or prospective surgical patient, will be invited to participate in a mixed-method usability evaluation to assess system effectiveness, system efficiency and user satisfaction (see Box 1). To complete usability evaluation, a process map will be created to define the number and type of task required to complete the measurement system.

Box 1 Definition of usability concepts

System effectiveness: the ability of participants to perform tasks to achieve pre-determined goals completely and accurately, and without negative consequences (e.g. poor layout of the system interface leading to participants missing or accidentally selecting system options) [35–38].

System efficiency: the amount of participant resources required to achieve the pre-specified goals (e.g. system completion time) [38,39].

User satisfaction: is the subjective opinions of participants based on their experience interacting with the system [38]. This includes any subjective reports about likes, dislikes and recommendations for changes [35].

1.2.1 System effectiveness

For peer review only - http://bmjopen.bmj.com/site/about/guidelines.xhtml

For peer review only

One-to-one user testing sessions will be used to assess system effectiveness by evaluating task completion and error rates. Sessions will involve completing the automated system in a simulated environment, applying concurrent think aloud techniques [40–42]. A topic guide will be developed and will structure the testing session discussions.

Patient and public representatives will be invited to participate in online user testing sessions. They will be eligible if they are over the age of 18. Individuals from two patient experience panels (NBT, BTHFT) will be recruited through respective panel coordinators. Sampling will be purposive to maximise variation in geographical location, ethnicity, and sex and will aim to include individuals whose first language is not English.

User testing will be completed using a video-conferencing software (e.g. Zoom, MS Teams) and audio-recorded. Two researchers familiar with the automated system and trained in qualitative research will conduct the user testing sessions. Observational notes will be taken to collect further information about challenges or errors encountered during task completion [43,44].

Task completion rates will be calculated as percentage of tasks completed. Error rates will be calculated based on number of user errors encountered. User errors will be deviations or problems experienced that will interfere with successfully completing the task. Number and type of non-critical errors (successfully addressed by testers themselves following instructions from the observer) and critical errors (require the observer to intervene or take remedial actions) will be noted. Results will be presented using descriptive statistics.

Understanding of system effectiveness will be supplemented by analysis of response rates generated through feasibility work in 1.1.

1.2.2 System efficiency

System efficiency will be assessed by calculating task completion time and task efficiency. Task completion time is defined as the time participants took from the first activity (starting the survey by following the hyperlink) to the last activity (submission of the survey). Task efficiency is defined as the time spent to complete each task. Analyses will be based on those who completed the automated system and for whom first and last activity timestamps were available.

1.2.3 User satisfaction

For peer review only - http://bmjopen.bmj.com/site/about/guidelines.xhtml

One-to-one user interviews will be conducted to assess user satisfaction in-depth. Interviews will explore issues including ease of use/navigation, satisfaction with instructions, satisfaction with the visual display, ease of access, burden, and likelihood of using the system again. Barriers and facilitators to completing the measurement system will also be explored. A topic guide will be tested and refined and used to direct discussions.

A sub-set of eligible patients and participants of the user testing sessions will be invited to take part. A purposive sampling strategy will be adopted to ensure that insights are drawn from a range of perspectives. Sampling characteristics will be 1) experience with surgery (vascular, gastrointestinal, urological, neurosurgical, gynaecological, breast or orthopaedic surgery) and good/bad SDM experience, 2) sex, 3) age, 4) ethnicity and 5) individuals whose first language is not English. Participant characteristics will be assessed as the study progresses and recruitment efforts will focus to target under-represented patients as necessary. Recruitment of the subset of patient participants will be undertaken by the principal investigator, research nurse or clinical collaborators via email or telephone. User testing participants will be recruited by researchers during the user testing sessions and interviews will be conducted immediately following the user testing session.

Interviews will be conducted primarily remotely (e.g. telephone or video conference) by experienced and trained qualitative researchers. All audio-recorded interviews will be transcribed and anonymised. Transcripts will be thematically analysed (see Qualitative analysis section in 1.3).

1.3 Views of diverse stakeholders to inform the use of the system to improve SDM

Qualitative research with wider stakeholders (including patients, healthcare professionals and members of the community) will be conducted to understand views of multiple stakeholders to inform the use of the system to improve SDM. Opinions about the acceptability and potential impact of real-time monitoring of SDM will be sought. Views on potential intervention components (activities), mechanisms of change, intermediate outcomes, assumptions, and indicators will be explored. Results will be used to co-develop initial programme theory to inform phase 2.

Patients and members of the public and community over the age of 18 will be eligible to take part. The sample will include people who are disproportionately affected by a poor SDM process and outcomes of surgery: those that are economically disadvantaged, from minority ethnic groups, and in older age. Professionals working in participating Trusts will be eligible for inclusion and may include surgeons, anaesthetists, nurses, perioperative care physicians, allied health professionals and hospital managers.

For peer review only - http://bmjopen.bmj.com/site/about/guidelines.xhtml

For peer review only

Recruitment

Eligible participants will be identified through existing networks, collaborations with local hospital patient panels, community leaders, and patients who have participated in feasibility (1.1) and usability (1.2) data collection. We will seek to recruit individuals who experience multiple intersecting inequalities to ensure the views of those with barriers to accessing healthcare are incorporated [45]. Recruitment of members of the community will be conducted using techniques developed and successfully applied by the Born in Bradford team [46,47] and the patient and public involvement and engagement (PPIE) group of the NIHR Bristol BRC. Recruitment materials will be translated into most spoken languages within the local areas.

Purposive sampling will seek to achieve diversity in relation to socio-demographic characteristics (e.g. age, gender), experience with surgery or SDM (e.g. surgical specialty, good/bad SDM experience) or under-served groups (economically disadvantaged, older age, ethnic minority). Where appropriate, snowball sampling will also be used, whereby individuals who participate in the study are asked about other potentially interested participants. The sample size will ultimately depend on theoretical saturation (i.e. when no new insights are identified from the data and sufficient data are collected to address the research question) [48,49]. It is anticipated that approximately 130 participants (around 105 patients and members of the community, and 25 professionals) will be required.

Data collection

Data collection will apply a flexible strategy to minimise perception that the research is 'hard-to-engage-with' [46]. A range of qualitative research methods are planned remotely and/or face-to-face including: i) semi-structured interviews, ii) focus groups and iii) participatory approaches (e.g. community events, discussion groups). It is anticipated that a minimum of 30 one-to-one interviews and six focus groups are required, complemented by recruitment through community events and discussion groups. However, these methods may be adapted based on evolving best-practice evidence from citizen science [50] and feedback from PPIE stakeholders. For example, evidence suggests that some British Asian people may be more willing to participate in a focus group in a familiar setting (e.g. community centre) than other settings [51].

Interviews and focus groups will be facilitated by experienced qualitative researchers based in Bristol and Bradford. Topic guides for interviews and focus groups will be developed to direct discussions. This will be iteratively refined during data collection to explore emergent views. Interviews and focus

For peer review only - http://bmjopen.bmj.com/site/about/guidelines.xhtml

groups will be held face-to-face, over the telephone or using a secure video conference service (e.g. Zoom MS, Teams,) but will ultimately depend on participant preference. Data collection will primarily be conducted in English. However, where data are collected from non-English speaking members of the community, additional support will be provided by interpreters and specialist researchers who conduct relevant foreign language interviews and focus groups. All interviews and focus groups will be audio recorded and transcribed verbatim. Field notes will be taken during the interviews.

Qualitative analyses

Transcripts and field notes will be analysed using a thematic approach with the help of a qualitive data management software (NVivo). Principles of thematic analysis will be applied to the data whereby (i) transcripts and notes will be read and re-read, (ii) codes are generated and assigned to relevant excerpts within the transcripts, (iii) themes will be identified by collating similar codes, (iv) accuracy of themes will be checked and (v) detailed analysis of themes will take place [52]. Analysis will involve linking transcripts and observational notes by integrating relevant data from both sources to gain a more comprehensive understanding of key findings. This process will primarily be inductive, with codes developed and iteratively refined through interpretation of the data. There will, however, be an a priori interest in examining data in relation to the study aims. For example, information to support evidence for the acceptability of monitoring the SDM process, and impact of monitoring on clinical care will actively be sought.

Analyses will be conducted separately for different stakeholder groups (patients, professionals, community) to help ascertain different viewpoints or experiences reported by each participant group. Depending on findings, an additional layer of analysis may be conducted to contrast results for several sub-groups (e.g. different under-served groups; different specialties) to ensure differing perspectives and experiences by population and context are accounted for in later intervention development. At least two experienced qualitative researchers will perform analysis independently and meet regularly to discuss impressions of the data. A subset of transcripts will be double coded by another experienced qualitative researcher. Any discrepancies in coding or interpretation of data will be referred to the wider study team for further discussions.

Summaries of findings from the analyses (descriptive reports) will be written, combining preliminary findings from the various data sources in relation to the study objectives. Drafts of these summaries will be prepared following rounds of recruitment and analyses and discussed within the study team.

For peer review only - http://bmjopen.bmj.com/site/about/guidelines.xhtml

For peer review only

The summaries will be iteratively developed as analysis proceeds and will inform discussions about saturation.

Dedicated multi-disciplinary meetings involving public contributors will be held to articulate an initial programme theory to inform the future development of the intervention to be more inclusive of recognised under-served groups. A summary of key findings from qualitative data collection in 1.3. will be prepared. We will draw on behavioural (COM-B) [53] and organisational (Normalisation Process Theory) [54] change theory to identify theory of how the intervention will work for under-served groups. Summaries will be combined to form a comprehensive report, providing a basis for phase 2.

Data management

All data will be generated and handled in accordance with relevant directives and regulations (e.g. Data Protection Act 2018). Any data collected as part of qualitative data collection will be recorded using encrypted devices. Audio files will be securely transferred and transcribed by transcription services approved by the University of Bristol. Transcripts will ensure anonymity of participants (e.g. in future study outputs) by assigning pseudonyms or participant IDs to replace any names or identifiable information. All electronic data files will be saved in restricted folders only accessible to the research team, on secure University of Bristol network space that adheres to the University of Bristol's data security policies. Files containing any personal information (e.g. contact details) will exclusively use the linked participant ID and will be encrypted and stored securely on the university servers.

Study steering group

A dedicated study steering group will be convened to provide oversight and strategic direction for the study. It will include patients and independent clinical and methodological experts and will meet six monthly to review progress and provide strategic guidance.

Patient and public involvement and engagement

For peer review only - http://bmjopen.bmj.com/site/about/guidelines.xhtml

For peer review only

PPIE is central to the project and will play a key role throughout. Patient partners have helped define the research questions and draft the protocol. A PPIE strategy has been developed in collaboration with patient partners in the planning stages of this study to ensure it meets the needs of patients. It includes PPIE activities across 1) strategy and oversight, 2) study conduct and 3) dissemination. Involvement of the patient co-author (VS), a patient advisory group consisting of a members from a diverse background and patient representatives on our steering group will ensure the study focuses on patient needs throughout. PPIE activities will co-ordinate by an experienced researcher and will be evaluated. Any feedback will be used to iteratively evolve the PPIE strategy to meet the needs of advancing PPIE practices.

Ethics and dissemination

This study is part of a project spanning quality improvement and research. It is therefore subject to two governance processes requiring separate approvals: Approval to monitor patients' experience of SDM in routine clinical practice was initially approved through a quality improvement proposal at North Bristol NHS Trust (reference: Q80008). This was then incorporated into a larger programme of work, where all processes were approved through the appropriate governance framework (Consent & SDM Programme Board, reporting to an Executive Assurance Committee).

Ethical approval required to conduct interviews with NHS patients and professionals was granted by the NHS HRA North West - Liverpool Central Research Ethics Committee (reference: 21/PR/0345).

The results of this work will be presented to professionals (at conferences, as journal articles), shared with the public (social media, engagement events) and those who participated in the project. We will collaborate with organisations involved in SDM (National Institute for Health and Care Excellence, NHS England) to share findings from the study and maximise the value of our work. Materials produced for dissemination will be tailored to the target audience and will include plain summaries in various languages, formal and informal presentations, infographics, or posters.

REFERENCES

1 Bomhof-Roordink H, Gärtner FR, Stiggelbout AM, *et al.* Key components of shared decision making models: A systematic review. BMJ Open. 2019;**9**. doi:10.1136/bmjopen-2019-031763

2 Department of Health and Social Care. The NHS Constitution for England. 2021.https://www.gov.uk/government/publications/the-nhs-constitution-for-england/the-

For peer review only - http://bmjopen.bmj.com/site/about/guidelines.xhtml

nhs-constitution-for-england#introduction-to-the-nhs-constitution (accessed 15 Sep 2022).

3 National Health Service. The NHS Long Term Plan. Published Online First: 2019.www.longtermplan.nhs.uk (accessed 15 Sep 2022).

4 Archive BLUW. Health and Social Care Act 2012 - NHS Reforms. 2013.https://www.webarchive.org.uk/en/ukwa/collection/9 (accessed 11 May 2023).

5 Royal College of Surgeons. Good Surgical Practice. 2013. https://www.rcseng.ac.uk/standards-and-research/gsp/ (accessed 15 Sep 2022).

6 General Medical Council. Decision making and consent - ethical guidance. 2020. https://www.gmc-uk.org/ethical-guidance/ethical-guidance-for-doctors/decision-making-and-consent (accessed 15 Sep 2022).

7 Joseph-Williams N, Lloyd A, Edwards A, *et al.* Implementing shared decision making in the NHS: lessons from the MAGIC programme. *BMJ* 2017;**357**:j1744. doi:10.1136/bmj.j1744

8 Lagarde SM, Franssen SJ, Van Werven JR, *et al.* Patient preferences for the disclosure of prognosis after esophagectomy for cancer with curative intent. *Ann Surg Oncol* 2008;**15**:3289–98. doi:10.1245/s10434-008-0068-y

9 Légaré F, Adekpedjou R, Stacey D, *et al.* Interventions for increasing the use of shared decision making by healthcare professionals. *Cochrane database Syst Rev* 2018;**7**:CD006732. doi:10.1002/14651858.CD006732.PUB4

10 Snijders HS, Kunneman M, Bonsing BA, *et al.* Preoperative risk information and patient involvement in surgical treatment for rectal and sigmoid cancer. *Color Dis* 2014;**16**:O43–9. doi:10.1111/CODI.12481

11 Abbott TEF, Fowler AJ, Dobbs TD, *et al.* Frequency of surgical treatment and related hospital procedures in the UK: a national ecological study using hospital episode statistics. *BJA Br J Anaesth* 2017;**119**:249–57. doi:10.1093/BJA/AEX137

12 Care Quality Commission. Better care in my hands. 2016. https://www.cqc.org.uk/sites/default/files/20160519_Better_care_in_my_hands_FINAL.pdf%0Ahttp://www.cqc.org.uk/content/better-care-my-hands-review-how-people-are-involved-their-care

13 Elwyn G, Frosch DL, Kobrin S. Implementing shared decision-making: Consider all the consequences. *Implement Sci* 2016;**11**:1–10. doi:10.1186/S13012-016-0480-9/TABLES/2

14 Hughes TM, Merath K, Chen Q, *et al.* Association of shared decision-making on patient-reported health outcomes and healthcare utilization. *Am J Surg* 2018;**216**:7–12. doi:10.1016/J.AMJSURG.2018.01.011

15 Arterburn D, Wellman R, Westbrook E, *et al.* Introducing Decision Aids At Group Health Was Linked To Sharply Lower Hip And Knee Surgery Rates And Costs. *https://doi.org/101377/hlthaff20110686* 2017;**31**:2094–104. doi:10.1377/HLTHAFF.2011.0686

16 Kirsch M, Brown S, Smith BW, *et al.* The Presence and Persistence of Unrealistic Expectations in Patients Undergoing Nerve Surgery. *Neurosurgery* 2020;**86**:778–82. doi:10.1093/NEUROS/NYZ335

17 Homer CV, Tod AM, Thompson AR, *et al.* Expectations and patients' experiences of obesity prior to bariatric surgery: a qualitative study. *BMJ Open* 2016;**6**:e009389. doi:10.1136/BMJOPEN-2015-009389

For peer review only - http://bmjopen.bmj.com/site/about/guidelines.xhtml

18 Heisler M, Bouknight RR, Hayward RA, *et al.* The Relative Importance of Physician Communication, Participatory Decision Making, and Patient Understanding in Diabetes Self-management. *J Gen Intern Med* 2002;**17**:243–52. doi:10.1046/J.1525-1497.2002.10905.X

19 National Institute for Health and Care Excellence. Shard decision making [NG197]. Evidence reviews 2021. NICE 2021. https://www.nice.org.uk/guidance/ng197/evidence/b-interventions-to-support-effective-shared-decision-making-pdf-9142344255 (accessed 15 Sep 2022).

20 Skivington K, Matthews L, Simpson SA, *et al.* A new framework for developing and evaluating complex interventions: update of Medical Research Council guidance. *BMJ* 2021;**374**:n2061. doi:10.1136/BMJ.N2061

21 Tong A, Sainsbury P, Craig J. Consolidated criteria for reporting qualitative research (COREQ): A 32-item checklist for interviews and focus groups. *Int J Qual Heal Care* 2007;**19**:349–57. doi:10.1093/intqhc/mzm042

22 Elwyn G, Frosch D, Thomson R, *et al.* Shared decision making: A model for clinical practice. J. Gen. Intern. Med. 2012;**27**:1361–7. doi:10.1007/s11606-012-2077-6

23 Joseph-Williams N, Williams D, Wood F, *et al.* A descriptive model of shared decision making derived from routine implementation in clinical practice ('Implement-SDM'): Qualitative study. *Patient Educ Couns* 2019;**102**:1774–85. doi:10.1016/j.pec.2019.07.016

24 National Institute for Health and Care Excellence. Shared decision making (NG197). 2021.

25 Care Quality Commission. North Bristol NHS Trust Inspection Report. 2019.

26 Gärtner FR, Bomhof-Roordink H, Smith IP, *et al.* The quality of instruments to assess the process of shared decision making: A systematic review. PLoS One. 2018;**13**. doi:10.1371/journal.pone.0191747

27 NHS England and NHS Improvement. Personalised Care Shared Decision Making Summary guide. 2019. https://www.england.nhs.uk/wp-content/uploads/2019/01/shared-decision-making-summary-guide-v1.pdf (accessed 26 Jan 2023).

28 NHS England Personalised Care. Shared Decision Making - Summary guide. https://www.england.nhs.uk/shared-decision-making/guidance-and-resources/ (accessed 15 Sep 2022).

29 Barr PJ, Forcino RC, Thompson R, *et al.* Evaluating CollaboRATE in a clinical setting: analysis of mode effects on scores, response rates and costs of data collection. *BMJ Open* 2017;**7**:e014681. doi:10.1136/BMJOPEN-2016-014681

30 Elwyn G, Barr PJ, Grande SW, *et al.* Developing CollaboRATE: A fast and frugal patient-reported measure of shared decision making in clinical encounters. *Patient Educ Couns* 2013;**93**:102–7. doi:10.1016/J.PEC.2013.05.009

31 Scholl I, Loon MK Van, Sepucha K, *et al.* Measurement of shared decision making – a review of instruments. *Z Evid Fortbild Qual Gesundhwes* 2011;**105**:313–24. doi:10.1016/J.ZEFQ.2011.04.012

32 Kriston L, Scholl I, Hölzel L, *et al.* The 9-item Shared Decision Making Questionnaire (SDM-Q-9). Development and psychometric properties in a primary care sample. *Patient Educ Couns* 2010;**80**:94–9. doi:10.1016/J.PEC.2009.09.034

33 Durand MA, Bekker HL, Casula A, *et al.* Can we routinely measure patient involvement in treatment decision-making in chronic kidney care? A service evaluation in 27 renal units in

For peer review only - http://bmjopen.bmj.com/site/about/guidelines.xhtml

the UK. *Clin Kidney J* 2016;**9**:252–9. doi:10.1093/CKJ/SFW003

34 Watson AC. Shared decision making and self-management support: Tools for empowering individuals to manage their health. *Prof Case Manag* 2015;**20**:103–5. doi:10.1097/NCM.0000000000000085

35 Bevan N, Carter J, Earthy J, *et al.* New ISO Standards for Usability, Usability Reports and Usability Measures. *Lect Notes Comput Sci (including Subser Lect Notes Artif Intell Lect Notes Bioinformatics)* 2016;**9731**:268–78. doi:10.1007/978-3-319-39510-4_25

36 ISO. ISO 9241-11:2018(en), Ergonomics of human-system interaction — Part 11: Usability: Definitions and concepts. 2018;:9241(11).https://www.iso.org/obp/ui/#iso:std:iso:9241:-11:ed-2:v1:en (accessed 24 Nov 2021).

37 Kyte D, Anderson N, Auti R, *et al.* Development of an electronic patient-reported outcome measure (ePROM) system to aid the management of patients with advanced chronic kidney disease. *J Patient-Reported Outcomes* 2020;**4**. doi:10.1186/s41687-020-00223-8

38 Aiyegbusi OL. Key methodological considerations for usability testing of electronic patient-reported outcome (ePRO) systems. *Qual Life Res* 2020;**29**:325–33. doi:10.1007/s11136-019-02329-z

39 Barnum CM. Establishing the essentials. In: Barnum CM, ed. *Usability testing essentials*. Boston: : Morgan Kaufmann 2011. 9–23.https://scholar.google.com/scholar?hl=en&as_sdt=0%2C5&q=Barnum%2C+C.+M.+%282011%29.+Establishing+the+essentials&btnG= (accessed 27 Jan 2023).

40 van den Haak MJ, de Jong MDT, Schellens PJ. Evaluation of an Informational Web Site: Three Variants of the Think-aloud Method Compared. *Tech Commun* 2007;**54**:58–71.

41 Alshammari T, Alhadreti O, Mayhew P. When to ask participants to think aloud:A comparative study of concurrent and retrospective think-aloud methods. *Int J Hum Comput Interact* 2015;**6**:48–64.

42 Khajouei R, Farahani F. A combination of two methods for evaluating the usability of a hospital information system. *BMC Med Inform Decis Mak* 2020;**20**:1–10. doi:10.1186/S12911-020-1083-6/TABLES/4

43 Farzanfar R, Finkelstein J, Friedman RH. Testing the Usability of Two Automated Home-Based Patient-Management Systems. *J Med Syst 2004 282* 2004;**28**:143–53. doi:10.1023/B:JOMS.0000023297.50379.3C

44 Kaufman DR, Patel VL, Hilliman C, *et al.* Usability in the real world: assessing medical information technologies in patients' homes. *J Biomed Inform* 2003;**36**:45–60. doi:10.1016/S1532-0464(03)00056-X

45 Chen JC, Obeng-Gyasi S. Intersectionality and the Surgical Patient: Expanding the Surgical Disparities Framework. *Ann Surg* 2022;**275**:E3–5. doi:10.1097/SLA.0000000000005045

46 Islam S, Joseph O, Chaudry A, *et al.* "We are not hard to reach, but we may find it hard to trust" …. Involving and engaging 'seldom listened to' community voices in clinical translational health research: a social innovation approach. *Res Involv Engagem 2021 71* 2021;**7**:1–15. doi:10.1186/S40900-021-00292-Z

47 Dickerson J, Bird PK, Bryant M, *et al.* Integrating research and system-wide practice in public health: Lessons learnt from Better Start Bradford. *BMC Public Health* 2019;**19**:1–12. doi:10.1186/S12889-019-6554-2/TABLES/8

For peer review only - http://bmjopen.bmj.com/site/about/guidelines.xhtml

48 Saunders B, Sim J, Kingstone T, *et al.* Saturation in qualitative research: exploring its conceptualization and operationalization. *Qual Quant* 2018;**52**:1893. doi:10.1007/S11135-017-0574-8

49 Hennink MM, Kaiser BN, Marconi VC. Code Saturation Versus Meaning Saturation: How Many Interviews Are Enough? *Qual Health Res* 2017;**27**:591–608. doi:10.1177/1049732316665344

50 Vohland K, Land-Zandstra A, Ceccaroni L, *et al. The Science of Citizen Science*. Cham: : Springer 2021. doi:10.1007/978-3-030-58278-4

51 Humm C, Schrögel P. Science for All? Practical Recommendations on Reaching Underserved Audiences. *Front Commun* 2020;**5**:42. doi:10.3389/FCOMM.2020.00042/BIBTEX

52 Braun V, Clarke V. *Successful qualitative research : a practical guide for beginners*. SAGE 2013.

53 Michie S, van Stralen MM, West R. The behaviour change wheel: A new method for characterising and designing behaviour change interventions. *Implement Sci* 2011;**6**:1–12. doi:10.1186/1748-5908-6-42/TABLES/3

54 Murray E, Treweek S, Pope C, *et al.* Normalisation process theory: A framework for developing, evaluating and implementing complex interventions. *BMC Med* 2010;**8**:1–11. doi:10.1186/1741-7015-8-63/TABLES/3

55 Harris PA, Taylor R, Thielke R, *et al.* Research electronic data capture (REDCap)--a metadata-driven methodology and workflow process for providing translational research informatics support. *J Biomed Inform* 2009;**42**:377–81. doi:10.1016/J.JBI.2008.08.010

For peer review only

Author contributions

AM developed the original idea for this study along with KNLA and JB. CH and AM wrote the first draft of the manuscript, and all co-authors reviewed and critically appraised the manuscript. AM has overall responsibility with strategic oversight from JB. VS contributed to the PPIE strategy. All authors read and approved the final version.

Funding statement

This project is funded by the National Institute for Health and Care Research (NIHR) Programme Development Grant (Co-Chief Investigators: AM & JB). It is also supported by an NIHR Clinician Scientist award to AM (NIHR CS-2017-17-010) and by the NIHR Biomedical Research Centre (BRC) at the University Hospitals Bristol and Weston NHS Foundation Trust and the University of Bristol (BRC-1215-20011). The views expressed in this publication are those of the authors and not necessarily

For peer review only - http://bmjopen.bmj.com/site/about/guidelines.xhtml

reflect those of the NIHR, the Department of Health and Social Care or the UK National Health Service.

Competing interests

All authors declare no competing interests.

Patient consent for publication

Participants subject to research governance approvals will provide electronic consent through a link to a secure data management platform (RedCap Version 11.1.18) [55] before any study activity will commence. As part of the consent process, participants will agree to their anonymised quotes being published in scientific journals.

Data availability statement

Relevant anonymised data will be included in published manuscripts and/or made available as supplementary files. Participant level data and the datasets generated and/or analysed during the current project will be stored in a non-publicly available repository. Pseudo-anonymised datasets will be made available via the University of Bristol's Research Data Repository, data.bris to bona fide researchers, subject to a legally binding data access agreement. Any applications to access data will involve a case-by-case review by the University of Bristol Data Access Committee. Qualifying researchers will be required to sign a data access agreement and closely liaise with study team members to ensure that the data they plan to make public are sufficiently anonymised. Generally, data will be made available for non-commercial use, only for the purpose of health and care research and with appropriate approvals in place.

For peer review only

For peer review only - http://bmjopen.bmj.com/site/about/guidelines.xhtml

BMJ Open

Protocol for a mixed-methods study to inform co-development of an inclusive intervention that uses real-time monitoring and feedback to improve shared decision making for surgery (the ALPACA study)

Journal:	*BMJ Open*
Manuscript ID	bmjopen-2023-079155.R1
Article Type:	Protocol
Date Submitted by the Author:	15-Nov-2023
Complete List of Authors:	Hoffmann, Christin; NIHR Bristol Biomedical Research Centre, Bristol Centre for Surgical Research, Bristol Medical School: Population Health Science, University of Bristol<br>Avery, Kerry; NIHR Bristol Biomedical Research Centre, Bristol Centre for Surgical Research, Bristol Medical School: Population Health Sciences, University of Bristol<br>Macefield, Rhiannon; NIHR Bristol Biomedical Research Centre, Bristol Centre for Surgical Research, Bristol Medical School: Population Health Science, University of Bristol<br>Snelgrove, Val; Patient representative<br>Blazeby, Jane; NIHR Bristol Biomedical Research Centre, Bristol Centre for Surgical Research, Bristol Medical School: Population Health Science, University of Bristol<br>Hopkins, Della; North Bristol NHS Trust<br>Hickey, Shireen; Bradford Teaching Hospitals NHS Foundation Trust<br>Cabral, Christie; University of Bristol Centre for Academic Primary Care, Bristol Medical School<br>Hall, Jennifer; Bradford Teaching Hospitals NHS Foundation Trust,<br>Gibbison, Ben; University of Bristol, ;<br>Rooshenas, Leila; NIHR Bristol Biomedical Research Centre, Bristol Centre for Surgical Research, Bristol Medical School: Population Health Science, University of Bristol<br>Williams, Adam; North Bristol NHS Trust Southmead Hospital<br>Aning, Jon; North Bristol NHS Trust, Bristol Urological Institute, Southmead Hospital; University of Bristol, Population Health Sciences, Bristol Medical School<br>Bekker, Hilary; University of Leeds, Leeds Unit of Complex Intervention Development (LUCID), Leeds Institute of Health Sciences, School of Medicine; Aarhus Universitet, The Research Centre for Patient Involvement (ResCenPI), Department of Public Health<br>McNair, Angus; University of Bristol, Centre for Surgical Research, Bristol Medical School: Population Health Sciences; North Bristol NHS Trust, GI Surgery<br>Judge, Andrew; University of Bristol, Musculoskeletal Research Unit<br>Smith, Andrew; North Bristol NHS Trust<br>Lingampalli, Archana; North Bristol NHS Trust<br>Reeves, Barnaby; University of Bristol, Bristol Trials Centre, Population Health Sciences, Bristol Medical School

	Preshaw, Jessica; North Bristol NHS Trust<br>Whitehouse, Michael; University of Bristol, Musculoskeletal Research Unit, Translational Health Sciences, Bristol Medical School; North Bristol NHS Trust<br>Cresswell, Paul; North Bristol NHS Trust<br>Braude, Philip; North Bristol NHS Trust<br>Potter, Shelley; University of Bristol, Translational Health Sciences, Bristol Medical School; North Bristol NHS Trust<br>Beckitt, Timothy; North Bristol NHS Trust<br>Whittlestone, Timothy; North Bristol NHS Trust

**Primary Subject Heading**:	Surgery
Secondary Subject Heading:	Patient-centred medicine, Qualitative research, Health services research, Communication
Keywords:	SURGERY, Decision Making, Patient Participation, Patient Reported Outcome Measures, Quality in health care < HEALTH SERVICES ADMINISTRATION & MANAGEMENT, QUALITATIVE RESEARCH

SCHOLARONE™
Manuscripts

Protocol for a mixed-methods study to inform co-development of an inclusive intervention that uses real-time monitoring and feedback to improve shared decision making for surgery (the ALPACA study).

Submission category: Protocol

Authors:
Christin Hoffmann[1] (Senior Research Associate in Health Services Research; BA, MSc, PhD),
Kerry NL Avery[1] (Associate Professor in Applied Health and Care Research; BSc, PhD),
Rhiannon C Macefield[1] (Research Fellow in Health Services Research; BSc, MSc, PhD),
Val Snelgrove[2] (Patient representative; HNC),
Jane M Blazeby[1] (Professor of Surgery; BSc, MB ChB, MD),
Della Hopkins[3] (Programme Manager; BSc, MSc, PhD),
Shireen Hickey[4] (Obstetrics and Gynaecology Registrar; MB ChB),
Christie Cabral[5] (Senior Research Fellow; BSc, MRes, PhD),
Jennifer Hall[4] (Senior Research Fellow; BSc, PhD),
Ben Gibbison[1,6] (Consultant Anaesthetist and Associate Professor in Cardiac Anaesthesia and Intensive Care; BSc, MMBS, MD),
Leila Rooshenas[1] (Associate Professor in Social Medicine and Health Policy; BSc, PhD),
Adam Williams[3] (Consultant Neurosurgeon; BSc, MB ChB),
Jonathan Aning[7, 8] (Consultant Urological Surgeon; DM, BM BS, BMedSci),
Hilary L Bekker[9, 10] (Professor of Medical Decision Making; CPsychol, BSc, MSc, PhD),
Angus GK McNair[1,3] (Consultant Colorectal Surgeon and Associate Professor in Colorectal Surgery; MB ChB, MRCS, PhD)

on behalf of the ALPACA study team:

Andy Judge[1] (Professor of Translational Statistics; MSc, PhD);
Andrew Smith[3] (Consultant Colorectal Surgeon; BSc, MBBS, MD);
Archana Lingampalli[3] (Business Intelligence; BA, PGDip)
Barnaby Reeves[11] (Professor of Health Services Research; BA, MSc, PhD);
Jessica Preshaw[3] (Consultant Gynaecologist; BSc, MB ChB);
Michael R Whitehouse[1,3] (Consultant Trauma and Orthopaedic Surgeon and Professor of Trauma and Orthopaedics; BSc, MB ChB, MSc, PGCert; PhD);
Paul Cresswell[3] (Director of Quality Governance; FCCA, MSc);
Philip Braude[3] (Consultant Geriatric Medicine; MBBS)
Shelley Potter[3] (Consultant Oncoplastic Breast Surgeon and Professor of Surgical Oncology; BSc, MB ChB, PhD)
Timothy Beckitt[3] (Consultant Vascular Surgeon; BSc MB ChB, MD, PGCert);
Timothy Whittlestone[3] (Medical Director and Consultant Urologist; MB BCh, MA, MD)

For peer review only - http://bmjopen.bmj.com/site/about/guidelines.xhtml

Author affiliations:

[1] National Institute for Health and Care Research Bristol Biomedical Research Centre, Bristol Centre for Surgical Research, Bristol Medical School: Population Health Sciences, University of Bristol, Bristol, BS8 2PS, UK

[2] Patient Representative

[3] North Bristol NHS Trust, Bristol, UK

[4] Bradford Institute for Health Research, Bradford Teaching Hospitals NHS Foundation Trust, Bradford, UK

[5] Centre for Academic Primary Care, Bristol Medical School, University of Bristol, Bristol, UK

[6] University Hospitals Bristol and Weston NHS Foundation Trust, Bristol, UK

[7] Bristol Urological Institute, Southmead Hospital, North Bristol Trust, Bristol, UK

[8] Population Health Sciences, Bristol Medical School, University of Bristol, Bristol, UK

[9] Leeds Unit of Complex Intervention Development (LUCID), Leeds Institute of Health Sciences, School of Medicine, University of Leeds, Leeds, UK

[10] The Research Centre for Patient Involvement (ResCenPI), Department of Public Health, Aarhus University, Central Denmark Region, DK

[11] Bristol Trials Centre, Population Health Sciences, Bristol Medical School, University of Bristol, Bristol, UK

Correspondence: Christin Hoffmann (c.hoffmann@bristol.ac.uk)

Running title: The ALPACA feasibility study protocol

Keywords: Shared decision making; Mixed-methods; Intervention development; Inequalities; Underserved groups; Outcome measurement; Protocol; Surgery; ePROMS; electronic measurement

Word count: 3,862

For peer review only

ABSTRACT

Introduction: High-quality shared decision making (SDM) is a priority of health services, but only achieved in a minority of surgical consultations. Improving SDM for surgical patients may lead to more effective care and moderate the impact of treatment consequences. There is a need to establish effective ways to achieve sustained and large-scale improvements in SDM for all patients whatever their background. The ALPACA study aims to develop, pilot, and evaluate a decision support intervention that uses real-time feedback of patients' experience of SDM to change patients' and healthcare professionals' decision-making processes before adult elective surgery and to improve patient and health service outcomes.

Methods and analysis: This protocol outlines a mixed-method study, involving diverse stakeholders (adult patients, healthcare professionals, members of the community) and three NHS trusts in England. Detailed methods for the assessment of the feasibility, usability, and stakeholder views of implementing a novel system to monitor the SDM process for surgery automatically and in real-time are described. The study will measure the SDM process using validated instruments (CollaboRATE, SDM-Q-9, SHARED-Q10) and will conduct semi-structured interviews and focus groups to examine 1) the feasibility of automated data collection, 2) the usability of the novel system, and 3) the views of diverse stakeholders to inform the use of the system to improve SDM. Future phases of this work will complete the development and evaluation of the intervention.

Ethics and dissemination: Ethical approval was granted by the NHS HRA North West - Liverpool Central Research Ethics Committee (reference: 21/PR/0345). Approval was also granted by North Bristol NHS Trust to undertake quality improvement work (reference: Q80008) overseen by the consent & SDM programme board and reporting to an Executive Assurance Committee.

For peer review only - http://bmjopen.bmj.com/site/about/guidelines.xhtml

Strengths and limitations of this study

- A mixed-methods study design will use a diverse representative sample of surgical patients from a range of NHS trusts to determine the feasibility of data collection, the usability of the novel system and understand views of diverse stakeholders to inform use of the system.
- Recruitment will focus on recognised under-served groups (economically disadvantaged, older age, ethnic minority) from Bristol and Bradford to maximise reach to an ethnically and socio-economically diverse population.
- The study uses three validated questionnaires to monitor SDM (CollaboRATE, SDM-Q-9, SHARED-Q10), including first use of the SHARED-Q10 measure in a surgical setting.
- This study excluded patients without capacity due to distinct requirements and guidance for consent and shared decision making processes in this population.

For peer review only

For peer review only - http://bmjopen.bmj.com/site/about/guidelines.xhtml

Introduction

Shared decision making (SDM) is a process where patients are supported to reach decisions in collaboration with health professionals [1]. Global and United Kingdom (UK) policy [2–4], professional and regulatory guidelines [5,6] recommend SDM in all healthcare settings. Making good decisions is particularly important for the five million people per year deciding to have surgery in the UK because, unlike many medical therapies, the effects are usually immediate and irreversible. Ensuring patients and surgeons have discussed accurate information about all options and their consequences, exchanged their reasoning about, and preferences for, each option, and agreed the treatment plan is essential to a good SDM process.

Evidence shows there is scope to improve SDM for surgery. A systematic review of 22 surgical studies found that only 36% of 13,176 patients perceived their consultation as shared [7]. Other systematic reviews show that surgeons underestimate patients' information needs [8], and patients do not receive desired information before surgery [9]. Major surgical risks go undisclosed [10], and patients report feeling uninformed [11] and want more involvement in decision-making [12]. The impact of these deficiencies is inadequately understood. It is thought that improving SDM processes may lead to more effective care through enhanced clinician-patient reasoning [13], thereby supporting treatment choices with greater benefit/harm ratios [8] and reducing overall use of health services [14,15]. High-quality SDM may also moderate the impact of treatment harms through more realistic treatment expectations [16,17] and improved self-management [18].

Guidelines for the implementation of SDM have been recently published by the National Institute for Health and Care Excellence (NICE) that includes best-evidence from a Cochrane review [9] and consultation with 454 stakeholders. It concluded that a combination of interventions to support organisations, clinicians, and patients are needed, but the evidence for these interventions is often poor [19]. Key priority areas were identified for future research, including generating evidence about how to: sustain SDM implementation at an organisation/health service level; measure the effectiveness of the SDM process for different contexts/settings/people; and ensure the SDM process is inclusive of people from diverse backgrounds (e.g. ethnic minorities, persons of lower health literacy or income backgrounds).

The ALPACA Study aims to address these deficiencies. We will develop, pilot, and evaluate a decision support intervention that uses real-time feedback of patient experiences of the SDM process to impact patient and professional decision-making processes before adult elective surgery and improve patient and health service outcomes. The intervention will include 1) efficient, real-time

For peer review only - http://bmjopen.bmj.com/site/about/guidelines.xhtml

evaluation of patient experiences of SDM at scale, 2) timely feedback of individual patient-reported experiences of SDM to care teams before surgery and 3) activities supporting meaningful change in patient and professional decision-making about surgery, individually and together.

This project aims to enable surgical teams to remedy deficiencies in the SDM process before surgery and thereby addresses NICE research priorities to detect such deficits reported by the patient. The intervention will be deliverable at scale to create sustained improvement in SDM through system-wide changes in decision-making processes facilitated by continuous patient-reported feedback. It will be co-created with patients with a focus on inclusivity of recognised under-served groups. Developing methods for efficient evaluation of the SDM process will make measurement of SDM outcomes more consistent and meaningful.

Aim and objectives

The overall aim of this project is to develop, pilot, and evaluate a decision support intervention that uses real-time feedback of patient experience of SDM to change patient and professional decision-making processes before adult elective surgery and improve patient and health service outcomes. There are three phases with the following objectives:

Phase 1: Assess the feasibility, usability and stakeholder views of implementing an automated system to monitor the SDM process for surgery in real-time.

Phase 2: Co-develop and refine the intervention with patients and professionals to understand how the intervention works, for whom, and in what context using findings from Phase 1.

Phase 3: Evaluate the effectiveness, cost-effectiveness, and implementation of the intervention to improve patient and health service outcomes in the English NHS.

This protocol describes phase 1. Details of subsequent phases which will complete the development (Phase 2) and evaluation of the intervention (Phase 3) will be described in future publications.

Methods

For peer review only - http://bmjopen.bmj.com/site/about/guidelines.xhtml

The project will employ mixed-methods to develop a complex intervention comprising multiple components that will impact a wide range of stakeholders and system processes. The overall aim to develop and evaluate the intervention will be conducted according to Medical Research Council (MRC) guidelines [20]. Phase 1 reported here is consistent with the MRC framework's feasibility phase, with consideration of the core elements critical for complex intervention research. Any qualitative elements will be reported in accordance with the consolidated criteria for reporting qualitative studies (COREQ) guidelines [21].

Conceptualisation

There is no unified definition of SDM. A systematic review identified 40 SDM models currently available with 53 different elements clustered in 24 overarching components [1]. Components present in more than half of models were: 'describe treatment options' (88% of models); 'make a decision' (75%); 'patient preferences' (68%); 'tailor information' (65%); 'deliberate' (58%); 'create choice awareness' (55%) and 'learn about the patient' (55%).

This study will conceptualise SDM using the 'Three Talk model' (2012) [22], later refined to 'Implement-SDM' (2019) [23]. This single-component model provides a guide for enhancing health professional communication to deliver SDM, and is the most highly referenced model (>1800 citations, Web of Science). It involves three key steps consistent with other models of SDM: 1) introduction of choice, 2) describing options and 3) helping patients explore preferences and make decisions. This was the chosen model for an NHS MAGIC programme [7] and is recommended in NICE guidelines [24].

Setting

Research will be conducted at three UK hospital trusts (North Bristol Trust/NBT, University Hospitals Bristol and Weston NHS Foundation Trust/UHBWFT, and Bradford Teaching Hospitals NHS Foundation Trust/BTHFT) alongside quality improvement programmes to improve SDM. NBT is one of the largest acute NHS trusts in the UK [25]. It provides a full range of acute clinical care for both local and regional clinical commissioning groups in South-West England. Specialised services are provided through NHS England, Welsh Health Boards and Welsh Specialist Commissioners. Services provided include elective and emergency gastrointestinal surgery, obstetrics and gynaecology, as well as specialist regional services in urology, neurosciences, trauma and orthopaedic and vascular surgery. One UHBWFT department is included as the South-West England regional cardiac surgical

For peer review only - http://bmjopen.bmj.com/site/about/guidelines.xhtml

centre. BTHFT is an acute Trust in the North of England with a full range of elective and emergency surgical services. Bristol and Bradford were purposively selected to maximise reach to ensure a diverse representative sample is included (e.g. 26.8% classed as Asian or Asian British, compared with 5.5% in Bristol).

Phase 1: Assess the feasibility, usability and stakeholder views of implementing an automated system to monitor the SDM process for surgery in real-time

Phase 1 will determine whether it is feasible and acceptable to monitor SDM processes for surgery automatically and in real-time using a novel electronic system. Objectives are to explore:

1.1 Feasibility of automated data collection
1.2 Usability of the electronic measurement system
1.3 Views of diverse stakeholders to inform the use of the system to improve SDM

Each objective comprises separate methods which are described in turn below. This phase is expected to continue until June 2025.

1.1 Feasibility of automated data collection

Feasibility assessment is designed to establish the feasibility of automated real-time evaluation of patient experiences of SDM at scale and will identify opportunities to optimise recruitment and data collection.

Participants

All patients over the age of 18 who have been booked for planned vascular, gastrointestinal, urological, neurosurgical, gynaecological, breast, cardiac and orthopaedic surgical procedures at participating hospitals will be eligible to participate. Surgical departments have been selected to be broadly representative of a diverse range of surgical specialties. Excluded will be patients under the age of 18, those without capacity to consent for medical procedures, or undergoing unplanned (emergency) surgery or endoscopic procedures. Data related to eligibility criteria are routinely collected through electronic patient record (EPR) systems.

For peer review only - http://bmjopen.bmj.com/site/about/guidelines.xhtml

Measurement of patient experience

Real-time measurement of patient experiences of the process of SDM will be facilitated by a secure, automated system procured through a third-party provider approved by NHS trusts. The system is a customisable off-the-shelf electronic patient-reported outcome measurement software and has previously been used for electronic data capture in other countries. Eligible patients will be identified through EPR using algorithms developed in collaboration with the software provider. Structured data queries will be designed to extract details of patients booked for eligible procedures. Queries will be designed to run automatically, securely transferring data from the hospital to the software provider daily to account for changes in scheduling. The automated system will send three validated SDM measurement instruments within one day after surgery booking (i.e. real-time baseline measurement). This timepoint in the decision making process was chosen as a pragmatic point in time to represent patients' cumulative experiences of SDM for surgery which may include discussions with surgeons, physicians, general practitioners, nurses, family, and friends. The selected measurement instruments will be operationalised into an online survey and administered via short messaging service (SMS) or email. A reciprocal data feed will securely return patients' survey responses immediately to the hospital data warehouse for secure storage (real-time analysis and feedback). Follow-up measures are sent within one day before surgery by either SMS or email (real-time follow-up measurement). A schematic of the process and intervention aims is illustrated in Figure 1.

Selection of three SDM measures was made through discussions within the study team and were informed by a systematic review of SDM measurement instruments using COSMIN (consensus-based standards for the selection of health measurement instruments) methods [26], national guidelines [19] and recommendations and use within NHS clinical practice [27–29]. The CollaboRATE instrument is a validated 3-item patient-reported measure assessing the extent of SDM experienced by patients [30]. Assessment of the instrument using COSMIN methods demonstrated acceptable discriminative validity, concurrent validity, intra-rater reliability and sensitivity to change [26]. It has been used in excess of 40 studies [31], including evaluations of quality improvement projects in surgery [32]. The SDM-Q-9 instrument is a validated 9-item patient-reported measure that evaluates their perceptions of involvement in the decision-making process [33]. It has been widely used in interventional studies and demonstrates good reliability, structural validity [26,33]. Systematic review evidence recommended use of SDM-Q-9 for surgery [34]. The SHARED-Q10 instrument is a 10-item patient-reported measure to assess patient perceptions of information provided, involvement in consultations, and agreement with the decision made [35]. This measure is included because it was developed, validated, and used in an NHS quality improvement programme [36,37]

For peer review only - http://bmjopen.bmj.com/site/about/guidelines.xhtml

and evaluates domains beyond patient perception of professional communication. Complete measurement instruments can be found in Supplemental File, Figures S1_S3.

Analysis

Feasibility of real-time monitoring will be evaluated by analysis of overall recruitment rate and response rates and time to response for the SDM measures at baseline and follow-up. Response rates will be presented as a number and percentage based on patients who completed the measures (e.g. completed all three items of the CollaboRATE instrument). Issues of equality, diversity and inclusion will be explored by examining the correlation between responders/non-responders and sociodemographic patient variables extracted from EPR.

Relationships between responders and non-responders and clinical and sociodemographic details will be explored using uni- and multivariable logistic regression. Included will be age, sex, ethnicity, index of multiple deprivation, and clinical and treatment parameters will be explored (e.g. operation (three-digit OPCS code), diagnosis (ICD10), date of booking, specialty, number of outpatient appointments in relevant specialty, number and length of hospital inpatient episode in relevant specialty). Index of multiple deprivation will be derived by Lower Layer Super Output Area (LSOAs) for individuals' postcode. All variables will be extracted from routinely collected data in EPR.

In addition, the study team will document any relevant technical, financial, administrative, and logistical observations throughout the study and pertinent challenges using shared electronic records (e.g. Microsoft Office suite). Any learning points will be descriptively summarised.

1.2 Usability of the electronic measurement system

Post-deployment usability testing will be conducted according to International Standards Organisation (ISO) standards for human-systems interaction (9241-11:2018) to evaluate the system's use in this context [38,39]. System users, defined as anyone who is a current or prospective surgical patient, will be invited to participate in a mixed-method usability evaluation to assess system effectiveness, system efficiency and user satisfaction (see Box 1). To complete usability evaluation, a process map will be created to define the number and type of task required to complete the measurement system.

Box 1 Definition of usability concepts

For peer review only - http://bmjopen.bmj.com/site/about/guidelines.xhtml

System effectiveness: the ability of participants to perform tasks to achieve pre-determined goals completely and accurately, and without negative consequences (e.g. poor layout of the system interface leading to participants missing or accidentally selecting system options) [38–41].

System efficiency: the amount of participant resources required to achieve the pre-specified goals (e.g. system completion time) [41,42].

User satisfaction: is the subjective opinions of participants based on their experience interacting with the system [41]. This includes any subjective reports about likes, dislikes and recommendations for changes [38].

1.2.1 System effectiveness

One-to-one user testing sessions will be used to assess system effectiveness by evaluating task completion and error rates. Sessions will involve completing the automated system in a simulated environment, applying concurrent think aloud techniques [43–45]. A topic guide will be developed and will structure the testing session discussions (Supplemental File 1, Table S1).

Patient and public representatives will be invited to participate in online user testing sessions. They will be eligible if they are over the age of 18. Individuals from two patient experience panels (NBT, BTHFT) will be recruited through respective panel coordinators. Sampling will be purposive to maximise variation in geographical location, ethnicity, and sex and will aim to include individuals whose first language is not English.

User testing will be completed using a video-conferencing software (e.g. Zoom, MS Teams) and audio-recorded. Two researchers familiar with the automated system and trained in qualitative research will conduct the user testing sessions. Observational notes will be taken to collect further information about challenges or errors encountered during task completion [46,47].

Task completion rates will be calculated as percentage of tasks completed. Error rates will be calculated based on number of user errors encountered. User errors will be deviations or problems experienced that will interfere with successfully completing the task. Number and type of non-critical errors (successfully addressed by testers themselves following instructions from the observer) and critical errors (require the observer to intervene or take remedial actions) will be noted. Results will be presented using descriptive statistics.

Understanding of system effectiveness will be supplemented by analysis of response rates generated through feasibility work in 1.1.

For peer review only - http://bmjopen.bmj.com/site/about/guidelines.xhtml

For peer review only

1.2.2 System efficiency

System efficiency will be assessed by calculating task completion time and task efficiency. Task completion time is defined as the time participants took from the first activity (starting the survey by following the hyperlink) to the last activity (submission of the survey). Task efficiency is defined as the time spent to complete each task. Analyses will be based on those who completed the automated system and for whom first and last activity timestamps were available.

1.2.3 User satisfaction

One-to-one user interviews will be conducted to assess user satisfaction in-depth. Interviews will explore issues including ease of use/navigation, satisfaction with instructions, satisfaction with the visual display, ease of access, burden, and likelihood of using the system again. Barriers and facilitators to completing the measurement system will also be explored. A topic guide will be tested and refined and used to direct discussions.

A sub-set of eligible patients and participants of the user testing sessions will be invited to take part. A purposive sampling strategy will be adopted to ensure that insights are drawn from a range of perspectives. Sampling characteristics will be 1) experience with surgery (vascular, gastrointestinal, urological, neurosurgical, gynaecological, breast or orthopaedic surgery) and good/bad SDM experience, 2) sex, 3) age, 4) ethnicity and 5) individuals whose first language is not English. Participant characteristics will be assessed as the study progresses and recruitment efforts will focus to target under-represented patients as necessary. Recruitment of the subset of patient participants will be undertaken by the principal investigator, research nurse or clinical collaborators via email or telephone. User testing participants will be recruited by researchers during the user testing sessions and interviews will be conducted immediately following the user testing session.

Interviews will be conducted primarily remotely (e.g. telephone or video conference) by experienced and trained qualitative researchers. All audio-recorded interviews will be transcribed and anonymised. Transcripts will be thematically analysed (see Qualitative analysis section in 1.3).

1.3 Views of diverse stakeholders to inform the use of the system to improve SDM

Qualitative research with wider stakeholders (including patients, healthcare professionals and members of the community) will be conducted to understand views of multiple stakeholders to

For peer review only - http://bmjopen.bmj.com/site/about/guidelines.xhtml

inform the use of the system to improve SDM. Opinions about the acceptability and potential impact of real-time monitoring of SDM will be sought. Views on potential intervention components (activities), mechanisms of change, intermediate outcomes, assumptions, and indicators will be explored. Results will be used to co-develop initial programme theory to inform phase 2.

Patients and members of the public and community over the age of 18 will be eligible to take part. The sample will include people who are disproportionately affected by a poor SDM process and outcomes of surgery: those that are economically disadvantaged, from minority ethnic groups, and in older age [48–51]. Professionals working in participating Trusts will be eligible for inclusion and may include surgeons, anaesthetists, nurses, perioperative care physicians, allied health professionals and hospital managers.

Recruitment

Eligible participants will be identified through existing networks, collaborations with local hospital patient panels, community leaders, and patients who have participated in feasibility (1.1) and usability (1.2) data collection. We will seek to recruit individuals who experience multiple intersecting inequalities to ensure the views of those with barriers to accessing healthcare are incorporated [48]. Recruitment of members of the community will be conducted using techniques developed and successfully applied by the Born in Bradford team [52,53] and the patient and public involvement and engagement (PPIE) group of the NIHR Bristol BRC. Recruitment materials will be translated into most spoken languages within the local areas.

Purposive sampling will seek to achieve diversity in relation to socio-demographic characteristics (e.g. age, gender), experience with surgery or SDM (e.g. surgical specialty, good/bad SDM experience) or under-served groups (economically disadvantaged, older age, ethnic minority). Where appropriate, snowball sampling will also be used, whereby individuals who participate in the study are asked about other potentially interested participants. The sample size will ultimately depend on theoretical saturation (i.e. when no new insights are identified from the data and sufficient data are collected to address the research question) [54,55]. It is anticipated that approximately 130 participants (around 105 patients and members of the community, and 25 professionals) will be required.

Data collection

Data collection will apply a flexible strategy to minimise perception that the research is 'hard-to-engage-with' [52]. A range of qualitative research methods are planned remotely and/or face-to-

For peer review only - http://bmjopen.bmj.com/site/about/guidelines.xhtml

face including: i) semi-structured interviews, ii) focus groups and iii) participatory approaches (e.g. community events, discussion groups). It is anticipated that a minimum of 30 one-to-one interviews and six focus groups are required, complemented by recruitment through community events and discussion groups. However, these methods may be adapted based on evolving best-practice evidence from citizen science [56] and feedback from PPIE stakeholders. For example, evidence suggests that some British Asian people may be more willing to participate in a focus group in a familiar setting (e.g. community centre) than other settings [57].

Interviews and focus groups will be facilitated by experienced qualitative researchers based in Bristol and Bradford. Topic guides for interviews and focus groups will be developed to direct discussions. This will be iteratively refined during data collection to explore emergent views. Interviews and focus groups will be held face-to-face, over the telephone or using a secure video conference service (e.g. Zoom MS, Teams,) but will ultimately depend on participant preference. Data collection will primarily be conducted in English. However, where data are collected from non-English speaking members of the community, additional support will be provided by interpreters and specialist researchers who conduct relevant foreign language interviews and focus groups. All interviews and focus groups will be audio recorded and transcribed verbatim. Field notes will be taken during the interviews.

Qualitative analyses

Transcripts and field notes will be analysed using a thematic approach with the help of a qualitive data management software (NVivo). Principles of thematic analysis will be applied to the data whereby (i) transcripts and notes will be read and re-read, (ii) codes are generated and assigned to relevant excerpts within the transcripts, (iii) themes will be identified by collating similar codes, (iv) accuracy of themes will be checked and (v) detailed analysis of themes will take place [58]. Analysis will involve linking transcripts and observational notes by integrating relevant data from both sources to gain a more comprehensive understanding of key findings. This process will primarily be inductive, with codes developed and iteratively refined through interpretation of the data. There will, however, be an a priori interest in examining data in relation to the study aims. For example, information to support evidence for the acceptability of monitoring the SDM process, and impact of monitoring on clinical care will actively be sought.

Analyses will be conducted separately for different stakeholder groups (patients, professionals, community) to help ascertain different viewpoints or experiences reported by each participant

For peer review only - http://bmjopen.bmj.com/site/about/guidelines.xhtml

group. Depending on findings, an additional layer of analysis may be conducted to contrast results for several sub-groups (e.g. different under-served groups; different specialties) to ensure differing perspectives and experiences by population and context are accounted for in later intervention development. At least two experienced qualitative researchers will perform analysis independently and meet regularly to discuss impressions of the data. A subset of transcripts will be double coded by another experienced qualitative researcher. Any discrepancies in coding or interpretation of data will be referred to the wider study team for further discussions.

Summaries of findings from the analyses (descriptive reports) will be written, combining preliminary findings from the various data sources in relation to the study objectives. Drafts of these summaries will be prepared following rounds of recruitment and analyses and discussed within the study team. The summaries will be iteratively developed as analysis proceeds and will inform discussions about saturation.

Dedicated multi-disciplinary meetings involving public contributors will be held to articulate an initial programme theory to inform the future development of the intervention to be more inclusive of recognised under-served groups. A summary of key findings from qualitative data collection in 1.3. will be prepared. We will draw on behavioural (COM-B) [59] and organisational (Normalisation Process Theory) [60] change theory to identify theory of how the intervention will work for under-served groups. Summaries will be combined to form a comprehensive report, providing a basis for phase 2.

Data management

All data will be generated and handled in accordance with relevant directives and regulations (e.g. Data Protection Act 2018). Any data collected as part of qualitative data collection will be recorded using encrypted devices. Audio files will be securely transferred and transcribed by transcription services approved by the University of Bristol. Transcripts will ensure anonymity of participants (e.g. in future study outputs) by assigning pseudonyms or participant IDs to replace any names or identifiable information. All electronic data files will be saved in restricted folders only accessible to the research team, on secure University of Bristol network space that adheres to the University of Bristol's data security policies. Files containing any personal information (e.g. contact details) will exclusively use the linked participant ID and will be encrypted and stored securely on the university servers.

For peer review only - http://bmjopen.bmj.com/site/about/guidelines.xhtml

Study steering group

A dedicated study steering group will be convened to provide oversight and strategic direction for the study. It will include patients and independent clinical and methodological experts and will meet six monthly to review progress and provide strategic guidance.

Patient and public involvement and engagement

PPIE is central to the project and will play a key role throughout. Patient partners have helped define the research questions and draft the protocol. A PPIE strategy has been developed in collaboration with patient partners in the planning stages of this study to ensure it meets the needs of patients. It includes PPIE activities across 1) strategy and oversight, 2) study conduct and 3) dissemination. Involvement of the patient co-author (VS), a patient advisory group consisting of a members from a diverse background and patient representatives on our steering group will ensure the study focuses on patient needs throughout. PPIE activities will co-ordinate by an experienced researcher and will be evaluated. Any feedback will be used to iteratively evolve the PPIE strategy to meet the needs of advancing PPIE practices.

Ethics and dissemination

This study is part of a project spanning quality improvement and research. It is therefore subject to two governance processes requiring separate approvals: Approval to monitor patients' experience of SDM in routine clinical practice was initially approved through a quality improvement proposal at North Bristol NHS Trust (reference: Q80008). This was then incorporated into a larger programme of work, where all processes were approved through the appropriate governance framework (Consent & SDM Programme Board, reporting to an Executive Assurance Committee). Patients will provide consent to participate in real-time monitoring through indicating their agreement with Terms and Conditions for the programme of work before completing the survey administered through the measurement system.

Ethical approval required to conduct qualitative data collection with NHS patients and professionals was granted by the NHS HRA North West - Liverpool Central Research Ethics Committee (reference:

For peer review only - http://bmjopen.bmj.com/site/about/guidelines.xhtml

21/PR/0345). Participants will provide written consent to participate in qualitative data collection before any research activity will commence. Consent will be obtained electronically through a link to a secure data management platform (RedCap Version 11.1.18). As part of the consent process, participants will agree to their anonymised quotes being published in scientific journals.

The results of this work will be presented to professionals (at conferences, as journal articles), shared with the public (social media, engagement events) and those who participated in the project. We will collaborate with organisations involved in SDM (National Institute for Health and Care Excellence, NHS England) to share findings from the study and maximise the value of our work. Materials produced for dissemination will be tailored to the target audience and will include plain summaries in various languages, formal and informal presentations, infographics, or posters.

Author contributions

AM developed the original idea for this study along with KNLA and JMB. AM, KNLA, CH, RCM, JMB, DH, SH, CC, JH, BG, LR, AW, JA, HB contributed to the development of the research question and objectives and were involved in the design of the study protocol. KNLA, RCM, CC, LR, JH, HB provide methodological expertise. CH and AM wrote the first draft of the manuscript, and all co-authors reviewed and critically appraised the manuscript. AM (guarantor) has overall responsibility for the content and project with strategic oversight from JMB. VS contributed to the PPIE strategy. Collaborators part of the ALPACA Study team (AJ, AS, AL, BR, JP, MRW, PC, PB, SP, TB, TW) provide clinical liaison and subject expertise that have shaped the study design. All collaborators have critically reviewed the study proposal. All authors read and approved the final version.

Funding statement

This project is funded by the National Institute for Health and Care Research (NIHR) Programme Development Grant (Co-Chief Investigators: AM & JB). It is also supported by an NIHR Clinician Scientist award to AM (NIHR CS-2017-17-010) and by the NIHR Biomedical Research Centre (BRC) at the University Hospitals Bristol and Weston NHS Foundation Trust and the University of Bristol (BRC-1215-20011). The views expressed in this publication are those of the authors and not necessarily reflect those of the NIHR, the Department of Health and Social Care or the UK National Health Service.

For peer review only - http://bmjopen.bmj.com/site/about/guidelines.xhtml

Competing interests

All authors declare no competing interests.

Data availability statement

Relevant anonymised data will be included in published manuscripts and/or made available as supplementary files. Participant level data and the datasets generated and/or analysed during the current project will be stored in a non-publicly available repository. Pseudo-anonymised datasets will be made available via the University of Bristol's Research Data Repository, data.bris to bona fide researchers, subject to a legally binding data access agreement. Any applications to access data will involve a case-by-case review by the University of Bristol Data Access Committee. Qualifying researchers will be required to sign a data access agreement and closely liaise with study team members to ensure that the data they plan to make public are sufficiently anonymised. Generally, data will be made available for non-commercial use, only for the purpose of health and care research and with appropriate approvals in place.

For peer review only

For peer review only - http://bmjopen.bmj.com/site/about/guidelines.xhtml

REFERENCES

1 Bomhof-Roordink H, Gärtner FR, Stiggelbout AM, *et al.* Key components of shared decision making models: A systematic review. BMJ Open. 2019;**9**. doi:10.1136/bmjopen-2019-031763

2 Department of Health and Social Care. The NHS Constitution for England. 2021.https://www.gov.uk/government/publications/the-nhs-constitution-for-england/the-nhs-constitution-for-england#introduction-to-the-nhs-constitution (accessed 15 Sep 2022).

3 National Health Service. The NHS Long Term Plan. Published Online First: 2019.www.longtermplan.nhs.uk (accessed 15 Sep 2022).

4 Archive BLUW. Health and Social Care Act 2012 - NHS Reforms. 2013.https://www.webarchive.org.uk/en/ukwa/collection/9 (accessed 11 May 2023).

5 Royal College of Surgeons. Good Surgical Practice. 2013. https://www.rcseng.ac.uk/standards-and-research/gsp/ (accessed 15 Sep 2022).

6 General Medical Council. Decision making and consent - ethical guidance. 2020. https://www.gmc-uk.org/ethical-guidance/ethical-guidance-for-doctors/decision-making-and-consent (accessed 15 Sep 2022).

7 Joseph-Williams N, Lloyd A, Edwards A, *et al.* Implementing shared decision making in the NHS: lessons from the MAGIC programme. *BMJ* 2017;**357**:j1744. doi:10.1136/bmj.j1744

8 Lagarde SM, Franssen SJ, Van Werven JR, *et al.* Patient preferences for the disclosure of prognosis after esophagectomy for cancer with curative intent. *Ann Surg Oncol* 2008;**15**:3289–98. doi:10.1245/s10434-008-0068-y

9 Légaré F, Adekpedjou R, Stacey D, *et al.* Interventions for increasing the use of shared decision making by healthcare professionals. *Cochrane database Syst Rev* 2018;**7**:CD006732. doi:10.1002/14651858.CD006732.PUB4

10 Snijders HS, Kunneman M, Bonsing BA, *et al.* Preoperative risk information and patient involvement in surgical treatment for rectal and sigmoid cancer. *Color Dis* 2014;**16**:O43–9. doi:10.1111/CODI.12481

11 Abbott TEF, Fowler AJ, Dobbs TD, *et al.* Frequency of surgical treatment and related hospital procedures in the UK: a national ecological study using hospital episode statistics. *BJA Br J Anaesth* 2017;**119**:249–57. doi:10.1093/BJA/AEX137

12 Care Quality Commission. Better care in my hands. 2016. https://www.cqc.org.uk/sites/default/files/20160519_Better_care_in_my_hands_FINAL.pdf %0Ahttp://www.cqc.org.uk/content/better-care-my-hands-review-how-people-are-involved-their-care

13 Elwyn G, Frosch DL, Kobrin S. Implementing shared decision-making: Consider all the consequences. *Implement Sci* 2016;**11**:1–10. doi:10.1186/S13012-016-0480-9/TABLES/2

14 Hughes TM, Merath K, Chen Q, *et al.* Association of shared decision-making on patient-reported health outcomes and healthcare utilization. *Am J Surg* 2018;**216**:7–12. doi:10.1016/J.AMJSURG.2018.01.011

15 Arterburn D, Wellman R, Westbrook E, *et al.* Introducing decision aids at group health was linked to sharply lower hip and knee surgery rates and costs. *Health Aff* 2012;**31**:2094–104. doi:10.1377/hlthaff.2011.0686

For peer review only

For peer review only - http://bmjopen.bmj.com/site/about/guidelines.xhtml

16 Kirsch M, Brown S, Smith BW, *et al.* The Presence and Persistence of Unrealistic Expectations in Patients Undergoing Nerve Surgery. *Neurosurgery* 2020;**86**:778–82. doi:10.1093/NEUROS/NYZ335

17 Homer CV, Tod AM, Thompson AR, *et al.* Expectations and patients' experiences of obesity prior to bariatric surgery: a qualitative study. *BMJ Open* 2016;**6**:e009389. doi:10.1136/BMJOPEN-2015-009389

18 Heisler M, Bouknight RR, Hayward RA, *et al.* The Relative Importance of Physician Communication, Participatory Decision Making, and Patient Understanding in Diabetes Self-management. *J Gen Intern Med* 2002;**17**:243–52. doi:10.1046/J.1525-1497.2002.10905.X

19 National Institute for Health and Care Excellence. Shard decision making [NG197]. Evidence reviews 2021. NICE 2021. https://www.nice.org.uk/guidance/ng197/evidence/b-interventions-to-support-effective-shared-decision-making-pdf-9142344255 (accessed 15 Sep 2022).

20 Skivington K, Matthews L, Simpson SA, *et al.* A new framework for developing and evaluating complex interventions: update of Medical Research Council guidance. *BMJ* 2021;**374**:n2061. doi:10.1136/BMJ.N2061

21 Tong A, Sainsbury P, Craig J. Consolidated criteria for reporting qualitative research (COREQ): A 32-item checklist for interviews and focus groups. *Int J Qual Heal Care* 2007;**19**:349–57. doi:10.1093/intqhc/mzm042

22 Elwyn G, Frosch D, Thomson R, *et al.* Shared decision making: A model for clinical practice. J. Gen. Intern. Med. 2012;**27**:1361–7. doi:10.1007/s11606-012-2077-6

23 Joseph-Williams N, Williams D, Wood F, *et al.* A descriptive model of shared decision making derived from routine implementation in clinical practice ('Implement-SDM'): Qualitative study. *Patient Educ Couns* 2019;**102**:1774–85. doi:10.1016/j.pec.2019.07.016

24 National Institute for Health and Care Excellence. Shared decision making (NG197). 2021.

25 Care Quality Commission. North Bristol NHS Trust Inspection Report. 2019.

26 Gärtner FR, Bomhof-Roordink H, Smith IP, *et al.* The quality of instruments to assess the process of shared decision making: A systematic review. *PLoS One* 2018;**13**. doi:10.1371/journal.pone.0191747

27 NHS England and NHS Improvement. Personalised Care Shared Decision Making Summary guide. 2019. https://www.england.nhs.uk/wp-content/uploads/2019/01/shared-decision-making-summary-guide-v1.pdf (accessed 26 Jan 2023).

28 NHS England Personalised Care. Shared Decision Making - Summary guide. https://www.england.nhs.uk/shared-decision-making/guidance-and-resources/ (accessed 15 Sep 2022).

29 Barr PJ, Forcino RC, Thompson R, *et al.* Evaluating CollaboRATE in a clinical setting: analysis of mode effects on scores, response rates and costs of data collection. *BMJ Open* 2017;**7**:e014681. doi:10.1136/BMJOPEN-2016-014681

30 Elwyn G, Barr PJ, Grande SW, *et al.* Developing CollaboRATE: A fast and frugal patient-reported measure of shared decision making in clinical encounters. *Patient Educ Couns* 2013;**93**:102–7. doi:10.1016/J.PEC.2013.05.009

31 Scholl I, Loon MK Van, Sepucha K, *et al.* Measurement of shared decision making – a review of instruments. *Z Evid Fortbild Qual Gesundhwes* 2011;**105**:313–24.

For peer review only - http://bmjopen.bmj.com/site/about/guidelines.xhtml

For peer review only

doi:10.1016/J.ZEFQ.2011.04.012

32 Brodney S, Fowler FJ, Barry MJ, *et al.* Comparison of Three Measures of Shared Decision-Making: SDM Process_4, CollaboRATE, and SURE Scales. *Med Decis Making* 2019;**39**:673. doi:10.1177/0272989X19855951

33 Kriston L, Scholl I, Hölzel L, *et al.* The 9-item Shared Decision Making Questionnaire (SDM-Q-9). Development and psychometric properties in a primary care sample. *Patient Educ Couns* 2010;**80**:94–9. doi:10.1016/J.PEC.2009.09.034

34 de Mik SML, Stubenrouch FE, Balm R, *et al.* Systematic review of shared decision-making in surgery. *Br J Surg* 2018;**105**:1721–30. doi:10.1002/bjs.11009

35 Durand MA, Bekker HL, Casula A, *et al.* Can we routinely measure patient involvement in treatment decision-making in chronic kidney care? A service evaluation in 27 renal units in the UK. *Clin Kidney J* 2016;**9**:252–9. doi:10.1093/CKJ/SFW003

36 NHS Rightcare. Measuring Shared Decision Making: A review of research evidence - A report for the Shared Decision Making programme in partnership with Capita Group Plc. London: 2012. https://www.england.nhs.uk/wp-content/uploads/2013/08/7sdm-report.pdf

37 NHS Rightcare. Your Health – Your Decision. London: 2013. https://aqua.nhs.uk/wp-content/uploads/2021/10/Your-Health-Your-Decision-Evaluation-Report.pdf

38 Bevan N, Carter J, Earthy J, *et al.* New ISO Standards for Usability, Usability Reports and Usability Measures. *Lect Notes Comput Sci (including Subser Lect Notes Artif Intell Lect Notes Bioinformatics)* 2016;**9731**:268–78. doi:10.1007/978-3-319-39510-4_25

39 ISO. ISO 9241-11:2018(en), Ergonomics of human-system interaction — Part 11: Usability: Definitions and concepts. 2018;:9241(11).https://www.iso.org/obp/ui/#iso:std:iso:9241:-11:ed-2:v1:en (accessed 24 Nov 2021).

40 Kyte D, Anderson N, Auti R, *et al.* Development of an electronic patient-reported outcome measure (ePROM) system to aid the management of patients with advanced chronic kidney disease. *J Patient-Reported Outcomes* 2020;**4**. doi:10.1186/s41687-020-00223-8

41 Aiyegbusi OL. Key methodological considerations for usability testing of electronic patient-reported outcome (ePRO) systems. *Qual Life Res* 2020;**29**:325–33. doi:10.1007/s11136-019-02329-z

42 Barnum CM. Establishing the essentials. In: Barnum CM, ed. *Usability testing essentials*. Boston: : Morgan Kaufmann 2011. 9–23.https://scholar.google.com/scholar?hl=en&as_sdt=0%2C5&q=Barnum%2C+C.+M.+%282011%29.+Establishing+the+essentials&btnG= (accessed 27 Jan 2023).

43 van den Haak MJ, de Jong MDT, Schellens PJ. Evaluation of an Informational Web Site: Three Variants of the Think-aloud Method Compared. *Tech Commun* 2007;**54**:58–71.

44 Alshammari T, Alhadreti O, Mayhew P. When to ask participants to think aloud:A comparative study of concurrent and retrospective think-aloud methods. *Int J Hum Comput Interact* 2015;**6**:48–64.

45 Khajouei R, Farahani F. A combination of two methods for evaluating the usability of a hospital information system. *BMC Med Inform Decis Mak* 2020;**20**:1–10. doi:10.1186/S12911-020-1083-6/TABLES/4

46 Farzanfar R, Finkelstein J, Friedman RH. Testing the Usability of Two Automated Home-Based Patient-Management Systems. *J Med Syst 2004 282* 2004;**28**:143–53.

For peer review only - http://bmjopen.bmj.com/site/about/guidelines.xhtml

doi:10.1023/B:JOMS.0000023297.50379.3C

47 Kaufman DR, Patel VL, Hilliman C, *et al.* Usability in the real world: assessing medical information technologies in patients' homes. *J Biomed Inform* 2003;**36**:45–60. doi:10.1016/S1532-0464(03)00056-X

48 Chen JC, Obeng-Gyasi S. Intersectionality and the Surgical Patient: Expanding the Surgical Disparities Framework. *Ann Surg* 2022;**275**:E3–5. doi:10.1097/SLA.0000000000005045

49 Durand MA, Carpenter L, Dolan H, *et al.* Do interventions designed to support shared decision-making reduce health inequalities? A systematic review and meta-analysis. *PLoS One* 2014;**9**. doi:10.1371/JOURNAL.PONE.0094670

50 Tarver WL, Haggstrom DA. The Use of Cancer-Specific Patient-Centered Technologies Among Underserved Populations in the United States: Systematic Review. *J Med Internet Res* 2019;**21**:e10256. doi:10.2196/10256

51 De Acedo Lizárraga MLS, De Acedo Baquedano MTS, Cardelle-Elawar M. Factors that affect decision making: Gender and age differences. *Int J Psychol Psychol Ther* 2007;**7**:381–91.https://www.redalyc.org/pdf/560/56070306.pdf (accessed 1 Nov 2023).

52 Islam S, Joseph O, Chaudry A, *et al.* "We are not hard to reach, but we may find it hard to trust" …. Involving and engaging 'seldom listened to' community voices in clinical translational health research: a social innovation approach. *Res Involv Engagem 2021 71* 2021;**7**:1–15. doi:10.1186/S40900-021-00292-Z

53 Dickerson J, Bird PK, Bryant M, *et al.* Integrating research and system-wide practice in public health: Lessons learnt from Better Start Bradford. *BMC Public Health* 2019;**19**:1–12. doi:10.1186/S12889-019-6554-2/TABLES/8

54 Saunders B, Sim J, Kingstone T, *et al.* Saturation in qualitative research: exploring its conceptualization and operationalization. *Qual Quant* 2018;**52**:1893. doi:10.1007/S11135-017-0574-8

55 Hennink MM, Kaiser BN, Marconi VC. Code Saturation Versus Meaning Saturation: How Many Interviews Are Enough? *Qual Health Res* 2017;**27**:591–608. doi:10.1177/1049732316665344

56 Vohland K, Land-Zandstra A, Ceccaroni L, *et al. The Science of Citizen Science*. Cham: : Springer 2021. doi:10.1007/978-3-030-58278-4

57 Humm C, Schrögel P. Science for All? Practical Recommendations on Reaching Underserved Audiences. *Front Commun* 2020;**5**:42. doi:10.3389/FCOMM.2020.00042/BIBTEX

58 Braun V, Clarke V. *Successful qualitative research : a practical guide for beginners*. SAGE 2013.

59 Michie S, van Stralen MM, West R. The behaviour change wheel: A new method for characterising and designing behaviour change interventions. *Implement Sci* 2011;**6**:1–12. doi:10.1186/1748-5908-6-42/TABLES/3

60 Murray E, Treweek S, Pope C, *et al.* Normalisation process theory: A framework for developing, evaluating and implementing complex interventions. *BMC Med* 2010;**8**:1–11. doi:10.1186/1741-7015-8-63/TABLES/3

For peer review only - http://bmjopen.bmj.com/site/about/guidelines.xhtml

Figures

Figure 1. Schematic of measurement process

For peer review only

For peer review only - http://bmjopen.bmj.com/site/about/guidelines.xhtml

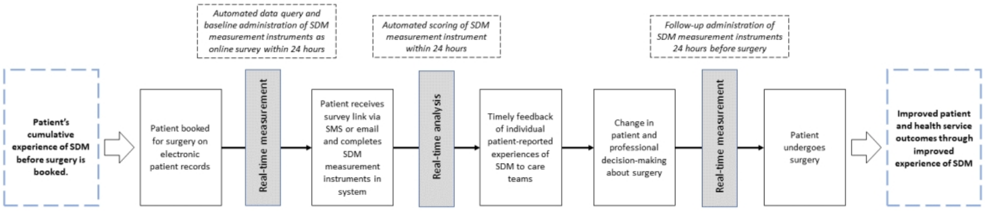

Figure 1. Schematic of measurement process

1086x378mm (38 x 38 DPI)

Supplemental File 1: The ALPACA Study

1. Instruments to measure the process of shared decision making

Figure S1 CollaboRATE measure

collaboRATE™

1. How much effort was made to help you understand your health issues?

0	1	2	3	4	5	6	7	8	9
No effort was made									Every effort was made

2. How much effort was made to listen to the things that matter most to you about your health issues?

0	1	2	3	4	5	6	7	8	9
No effort was made									Every effort was made

3. How much effort was made to include what matters most to you in choosing what to do next?

0	1	2	3	4	5	6	7	8	9
No effort was made									Every effort was made

From: Elwyn, Glyn, Paul James Barr, Stuart W. Grande, Rachel Thompson, Thom Walsh, and Elissa M. Ozanne. "Developing CollaboRATE: a fast and frugal patient-reported measure of shared decision making in clinical encounters." *Patient education and counseling* 93, no. 1 (2013): 102-107.

Figure S2 The 9-item Shared Decision Making Questionnaire (SDM-Q-9)

The 9-item Shared Decision Making Questionnaire (SDM-Q-9)

[Example] **Please indicate which health complaint/problem/illness the consultation was about:**

[Example] **Please indicate which decision was made:**

Nine statements related to the decision-making in your consultation are listed below. For each statement please indicate how much you agree or disagree.

	Statement	completely disagree	strongly disagree	somewhat disagree	somewhat agree	strongly agree	completely agree
**1.**	**My doctor made clear that a decision needs to be made.**	❑	❑	❑	❑	❑	❑
**2.**	**My doctor wanted to know exactly how I want to be involved in making the decision.**	❑	❑	❑	❑	❑	❑
**3.**	**My doctor told me that there are different options for treating my medical condition.**	❑	❑	❑	❑	❑	❑
**4.**	**My doctor precisely explained the advantages and disadvantages of the treatment options.**	❑	❑	❑	❑	❑	❑
**5.**	**My doctor helped me understand all the information.**	❑	❑	❑	❑	❑	❑
**6.**	**My doctor asked me which treatment option I prefer.**	❑	❑	❑	❑	❑	❑
**7.**	**My doctor and I thoroughly weighed the different treatment options.**	❑	❑	❑	❑	❑	❑
**8.**	**My doctor and I selected a treatment option together.**	❑	❑	❑	❑	❑	❑
**9.**	**My doctor and I reached an agreement on how to proceed.**	❑	❑	❑	❑	❑	❑

From: Kriston, L., Scholl, I., Hölzel, L., Simon, D., Loh, A., & Härter, M. (2010). The 9-item Shared Decision Making Questionnaire (SDM-Q-9). Development and psychometric properties in a primary care sample. *Patient education and counseling*, *80*(1), 94-99.

Figure S3 SHARED measure

SHARED – A Patient Experience of Shared Decision Making Questionnaire

What was your experience of making this treatment decision with your [health professional/s] – *for each statement, tick the box that matches best how much you agree with it:*

		Disagree Strongly	Disagree	Not Sure	Agree	Agree Strongly
	The [health] professional…					
**1**	talked about other options from the one I chose	☐	☐	☐	☐	☐
**2**	talked about whether or not there was a medically best option for my *[health problem]*	☐	☐	☐	☐	☐
**3**	gave their views about the options	☐	☐	☐	☐	☐
**4**	asked for my views about the options	☐	☐	☐	☐	☐
	I talked about…					
**5**	what was important to me about this decision	☐	☐	☐	☐	☐
**6**	why one option suited me better than another	☐	☐	☐	☐	☐
**7**	the risks and benefits of the options for me and my health	☐	☐	☐	☐	☐
	I felt…					
**8**	it would be OK to choose any option we talked about	☐	☐	☐	☐	☐
**9**	the decision made was the best one for me	☐	☐	☐	☐	☐
**10**	the *[health]* professional and I agreed which option was the best one for me	☐	☐	☐	☐	☐

From: Bekker HL, Légaré F, Nye A, Walker W. SHARED – A Patient Experience of Shared Decision Making Questionnaire. (2012). University of Leeds, UK

For peer review only

2. Example topic guides for qualitative data collection

Table S1 Usability testing (1.2) – interview guide

Intro

- Thank you and introduction
- Explanation of project (assess to what extent), ask if there are questions
- Ask for preferred mode for completing the survey (sms/email) and send the link
- Check if ok to audio record → *switch on recording*
- Explain purpose of the session:
    - We would like to test the survey that patients receive when booked in for surgery.
    - The focus is on **functionality**. It helps us make improvements to the process.
    - This session is **NOT** about the wording of questions, we are just interested in the usability
    - The text/email is a tester only, so the responses you give are not real
- Explain specific tasks:
    - There are two surveys. 8 steps (3 questions) for the first one, 20 steps (9 questions) for the second one.
    - We will
        - run through these steps and see how you get on with these
        - might feel a little unnatural but is important you tell me what you think and what you see, what is clear/unclear, what is easy/not straight forward or difficult to complete
    - Say where there is a problem, e.g. that you had to press twice to proceed
- Explain there will be questions at the end

Think-aloud exercise

Start with 8 steps of CollaboRATE

- Prompts if participant doesn't talk
    - Can you tell me what you currently see?
    - What are you going to do next?
    - What can you see now?
- Prompts to elicit views
    - Could you tell me what you think about Step X?
    - How do you feel about Step X?
    - What do you think about Step X?
    - How clear is Step X?
    - How easy is Step X?

Pause and ask follow-up questions

- "Having just completed the survey…"
    - How easy do you think is it to respond to the survey?
    - What do you think about the length of the survey?
    - What are your thoughts on the overall visual display? How visually appealing is the survey?
    - What would stop people from doing the survey? *Why?*
    - What issues can you think of people might encounter when completing the survey? *Why?*
    - What else would you change about how the survey is delivered? *Why?*

For peer review only

Table S2 Exploring views of under-served groups (1.3) – interview guide

Background

- Intro to interviewer (name, role and inability to answer care-related questions)
- Explain what SDM is and the main aim of the research project (focus on what SDM is vs isn't, current problem and relevance/importance to community)
- Reminder of anonymity, confidentiality and that interview can be stopped at any time.
- *Take questions*
- Reminder of recording and check participant is happy
- *Switch on recorder*

Explore context of SDM experience

Having just explained a bit more about what the research aims to do and what good shared decision making looks like, I am interested to hear from you...

- What is your experience with decision making for any healthcare treatment?
- How much involvement in decisions about surgical treatment would you prefer?
- Do you feel that, for whatever reason, you felt that you were/would be treated unequal to others in terms of making decision?

Exploring intervention components

- What do you think about the hospital using this survey to record people's experiences of how involved they felt in the surgical decision making?
- How do you feel about personal survey responses being passed on to the clinical team and you/the patient not being anonymous?
- What do you think needs to happen next to make sure you/ patients are given a voice and SDM is improved?
- How well do you think this process would work for everyone?

For peer review only

BMJ Open

Protocol for a mixed-methods study to inform co-development of an inclusive intervention that uses real-time monitoring and feedback to improve shared decision making for surgery (the ALPACA study)

Journal:	*BMJ Open*
Manuscript ID	bmjopen-2023-079155.R2
Article Type:	Protocol
Date Submitted by the Author:	05-Dec-2023

Complete List of Authors:	Hoffmann, Christin; NIHR Bristol Biomedical Research Centre, Bristol Centre for Surgical Research, Bristol Medical School: Population Health Science, University of Bristol<br>Avery, Kerry; NIHR Bristol Biomedical Research Centre, Bristol Centre for Surgical Research, Bristol Medical School: Population Health Sciences, University of Bristol<br>Macefield, Rhiannon; NIHR Bristol Biomedical Research Centre, Bristol Centre for Surgical Research, Bristol Medical School: Population Health Science, University of Bristol<br>Snelgrove, Val; Patient representative<br>Blazeby, Jane; NIHR Bristol Biomedical Research Centre, Bristol Centre for Surgical Research, Bristol Medical School: Population Health Science, University of Bristol<br>Hopkins, Della; North Bristol NHS Trust<br>Hickey, Shireen; Bradford Teaching Hospitals NHS Foundation Trust<br>Cabral, Christie; University of Bristol Centre for Academic Primary Care, Bristol Medical School<br>Hall, Jennifer; Bradford Teaching Hospitals NHS Foundation Trust,<br>Gibbison, Ben; University of Bristol, ;<br>Rooshenas, Leila; NIHR Bristol Biomedical Research Centre, Bristol Centre for Surgical Research, Bristol Medical School: Population Health Science, University of Bristol<br>Williams, Adam; North Bristol NHS Trust Southmead Hospital<br>Aning, Jon; North Bristol NHS Trust, Bristol Urological Institute, Southmead Hospital; University of Bristol, Population Health Sciences, Bristol Medical School<br>Bekker, Hilary; University of Leeds, Leeds Unit of Complex Intervention Development (LUCID), Leeds Institute of Health Sciences, School of Medicine; Aarhus Universitet, The Research Centre for Patient Involvement (ResCenPI), Department of Public Health<br>McNair, Angus; University of Bristol, Centre for Surgical Research, Bristol Medical School: Population Health Sciences; North Bristol NHS Trust, GI Surgery<br>Judge, Andrew; University of Bristol, Musculoskeletal Research Unit<br>Smith, Andrew; North Bristol NHS Trust<br>Lingampalli, Archana; North Bristol NHS Trust<br>Reeves, Barnaby; University of Bristol, Bristol Trials Centre, Population Health Sciences, Bristol Medical School

Preshaw, Jessica; North Bristol NHS Trust
Whitehouse, Michael; University of Bristol, Musculoskeletal Research Unit, Translational Health Sciences, Bristol Medical School; North Bristol NHS Trust
Cresswell, Paul; North Bristol NHS Trust
Braude, Philip; North Bristol NHS Trust
Potter, Shelley; University of Bristol, Translational Health Sciences, Bristol Medical School; North Bristol NHS Trust
Beckitt, Timothy; North Bristol NHS Trust
Whittlestone, Timothy; North Bristol NHS Trust

**Primary Subject Heading**:	Surgery
Secondary Subject Heading:	Patient-centred medicine, Qualitative research, Health services research, Communication
Keywords:	SURGERY, Decision Making, Patient Participation, Patient Reported Outcome Measures, Quality in health care < HEALTH SERVICES ADMINISTRATION & MANAGEMENT, QUALITATIVE RESEARCH

SCHOLARONE™
Manuscripts

Protocol for a mixed-methods study to inform co-development of an inclusive intervention that uses real-time monitoring and feedback to improve shared decision making for surgery (the ALPACA study).

Submission category: Protocol

Authors:
Christin Hoffmann[1] (Senior Research Associate in Health Services Research; BA, MSc, PhD),
Kerry NL Avery[1] (Associate Professor in Applied Health and Care Research; BSc, PhD),
Rhiannon C Macefield[1] (Research Fellow in Health Services Research; BSc, MSc, PhD),
Val Snelgrove[2] (Patient representative; HNC),
Jane M Blazeby[1] (Professor of Surgery; BSc, MB ChB, MD),
Della Hopkins[3] (Programme Manager; BSc, MSc, PhD),
Shireen Hickey[4] (Obstetrics and Gynaecology Registrar; MB ChB),
Christie Cabral[5] (Senior Research Fellow; BSc, MRes, PhD),
Jennifer Hall[4] (Senior Research Fellow; BSc, PhD),
Ben Gibbison[1,6] (Consultant Anaesthetist and Associate Professor in Cardiac Anaesthesia and Intensive Care; BSc, MMBS, MD),
Leila Rooshenas[1] (Associate Professor in Social Medicine and Health Policy; BSc, PhD),
Adam Williams[3] (Consultant Neurosurgeon; BSc, MB ChB),
Jonathan Aning[7, 8] (Consultant Urological Surgeon; DM, BM BS, BMedSci),
Hilary L Bekker[9, 10] (Professor of Medical Decision Making; CPsychol, BSc, MSc, PhD),
Angus GK McNair[1,3] (Consultant Colorectal Surgeon and Associate Professor in Colorectal Surgery; MB ChB, MRCS, PhD)

on behalf of the ALPACA study team:

Andy Judge[1] (Professor of Translational Statistics; MSc, PhD);
Andrew Smith[3] (Consultant Colorectal Surgeon; BSc, MBBS, MD);
Archana Lingampalli[3] (Business Intelligence; BA, PGDip)
Barnaby Reeves[11] (Professor of Health Services Research; BA, MSc, PhD);
Jessica Preshaw[3] (Consultant Gynaecologist; BSc, MB ChB);
Michael R Whitehouse[1,3] (Consultant Trauma and Orthopaedic Surgeon and Professor of Trauma and Orthopaedics; BSc, MB ChB, MSc, PGCert; PhD);
Paul Cresswell[3] (Director of Quality Governance; FCCA, MSc);
Philip Braude[3] (Consultant Geriatric Medicine; MBBS)
Shelley Potter[3] (Consultant Oncoplastic Breast Surgeon and Professor of Surgical Oncology; BSc, MB ChB, PhD)
Timothy Beckitt[3] (Consultant Vascular Surgeon; BSc MB ChB, MD, PGCert);
Timothy Whittlestone[3] (Medical Director and Consultant Urologist; MB BCh, MA, MD)

For peer review only - http://bmjopen.bmj.com/site/about/guidelines.xhtml

Author affiliations:

[1] National Institute for Health and Care Research Bristol Biomedical Research Centre, Bristol Centre for Surgical Research, Bristol Medical School: Population Health Sciences, University of Bristol, Bristol, BS8 2PS, UK

[2] Patient Representative

[3] North Bristol NHS Trust, Bristol, UK

[4] Bradford Institute for Health Research, Bradford Teaching Hospitals NHS Foundation Trust, Bradford, UK

[5] Centre for Academic Primary Care, Bristol Medical School, University of Bristol, Bristol, UK

[6] University Hospitals Bristol and Weston NHS Foundation Trust, Bristol, UK

[7] Bristol Urological Institute, Southmead Hospital, North Bristol Trust, Bristol, UK

[8] Population Health Sciences, Bristol Medical School, University of Bristol, Bristol, UK

[9] Leeds Unit of Complex Intervention Development (LUCID), Leeds Institute of Health Sciences, School of Medicine, University of Leeds, Leeds, UK

[10] The Research Centre for Patient Involvement (ResCenPI), Department of Public Health, Aarhus University, Central Denmark Region, DK

[11] Bristol Trials Centre, Population Health Sciences, Bristol Medical School, University of Bristol, Bristol, UK

Correspondence: Christin Hoffmann (c.hoffmann@bristol.ac.uk)

Running title: The ALPACA feasibility study protocol

Keywords: Shared decision making; Mixed-methods; Intervention development; Inequalities; Underserved groups; Outcome measurement; Protocol; Surgery; ePROMS; electronic measurement

Word count: 3,862

For peer review only

ABSTRACT

Introduction: High-quality shared decision making (SDM) is a priority of health services, but only achieved in a minority of surgical consultations. Improving SDM for surgical patients may lead to more effective care and moderate the impact of treatment consequences. There is a need to establish effective ways to achieve sustained and large-scale improvements in SDM for all patients whatever their background. The ALPACA study aims to develop, pilot, and evaluate a decision support intervention that uses real-time feedback of patients' experience of SDM to change patients' and healthcare professionals' decision-making processes before adult elective surgery and to improve patient and health service outcomes.

Methods and analysis: This protocol outlines a mixed-method study, involving diverse stakeholders (adult patients, healthcare professionals, members of the community) and three NHS trusts in England. Detailed methods for the assessment of the feasibility, usability, and stakeholder views of implementing a novel system to monitor the SDM process for surgery automatically and in real-time are described. The study will measure the SDM process using validated instruments (CollaboRATE, SDM-Q-9, SHARED-Q10) and will conduct semi-structured interviews and focus groups to examine 1) the feasibility of automated data collection, 2) the usability of the novel system, and 3) the views of diverse stakeholders to inform the use of the system to improve SDM. Future phases of this work will complete the development and evaluation of the intervention.

Ethics and dissemination: Ethical approval was granted by the NHS HRA North West - Liverpool Central Research Ethics Committee (reference: 21/PR/0345). Approval was also granted by North Bristol NHS Trust to undertake quality improvement work (reference: Q80008) overseen by the consent & SDM programme board and reporting to an Executive Assurance Committee.

For peer review only - http://bmjopen.bmj.com/site/about/guidelines.xhtml

Strengths and limitations of this study

- A mixed-methods study design will use a diverse representative sample of surgical patients from a range of NHS trusts to determine the feasibility of data collection, the usability of the novel system and understand views of diverse stakeholders to inform use of the system.
- Recruitment will focus on recognised under-served groups (economically disadvantaged, older age, ethnic minority) from Bristol and Bradford to maximise reach to an ethnically and socio-economically diverse population.
- The study uses three validated questionnaires to monitor SDM (CollaboRATE, SDM-Q-9, SHARED-Q10), including first use of the SHARED-Q10 measure in a surgical setting.
- This study excluded patients without decisional capacity due to distinct requirements and guidance for consent and shared decision making processes in this population.

For peer review only

For peer review only - http://bmjopen.bmj.com/site/about/guidelines.xhtml

Introduction

Shared decision making (SDM) is a process where patients are supported to reach decisions in collaboration with health professionals [1]. Global and United Kingdom (UK) policy [2–4], professional and regulatory guidelines [5,6] recommend SDM in all healthcare settings. Making good decisions is particularly important for the five million people per year deciding to have surgery in the UK because, unlike many medical therapies, the effects are usually immediate and irreversible. Ensuring patients and surgeons have discussed accurate information about all options and their consequences, exchanged their reasoning about, and preferences for, each option, and agreed the treatment plan is essential to a good SDM process.

Evidence shows there is scope to improve SDM for surgery. A systematic review of 22 surgical studies found that only 36% of 13,176 patients perceived their consultation as shared [7]. Other systematic reviews show that surgeons underestimate patients' information needs [8], and patients do not receive desired information before surgery [9]. Major surgical risks go undisclosed [10], and patients report feeling uninformed [11] and want more involvement in decision-making [12]. The impact of these deficiencies is inadequately understood. It is thought that improving SDM processes may lead to more effective care through enhanced clinician-patient reasoning [13], thereby supporting treatment choices with greater benefit/harm ratios [8] and reducing overall use of health services [14,15]. High-quality SDM may also moderate the impact of treatment harms through more realistic treatment expectations [16,17] and improved self-management [18].

Guidelines for the implementation of SDM have been recently published by the National Institute for Health and Care Excellence (NICE) that includes best-evidence from a Cochrane review [9] and consultation with 454 stakeholders. It concluded that a combination of interventions to support organisations, clinicians, and patients are needed, but the evidence for these interventions is often poor [19]. Key priority areas were identified for future research, including generating evidence about how to: 1) sustain SDM implementation at an organisation/health service level, 2) measure the effectiveness of the SDM process for different contexts/settings/people, and 3) ensure the SDM process is inclusive of people from diverse backgrounds (e.g. ethnic minorities, persons of lower health literacy or income backgrounds).

The ALPACA Study aims to address these deficiencies. We will develop, pilot, and evaluate a decision support intervention that uses real-time feedback of patient experiences of the SDM process to impact patient and professional decision-making processes before adult elective surgery and improve patient and health service outcomes. The intervention will include 1) efficient, real-time

For peer review only - http://bmjopen.bmj.com/site/about/guidelines.xhtml

evaluation of patient experiences of SDM at scale, 2) timely feedback of individual patient-reported experiences of SDM to care teams before surgery and 3) activities supporting meaningful change in patient and professional decision-making about surgery, individually and together.

This project aims to enable surgical teams to remedy deficiencies in the SDM process before surgery and thereby addresses NICE research priorities to detect such deficits reported by the patient. The intervention will be deliverable at scale to create sustained improvement in SDM through system-wide changes in decision-making processes facilitated by continuous patient-reported feedback. It will be co-created with patients with a focus on inclusivity of recognised under-served groups. Developing methods for efficient evaluation of the SDM process will make measurement of SDM outcomes more consistent and meaningful.

Aim and objectives

The overall aim of this project is to develop, pilot, and evaluate a decision support intervention that uses real-time feedback of patient experience of SDM to change patient and professional decision-making processes before adult elective surgery and improve patient and health service outcomes. There are three phases with the following objectives:

Phase 1: Assess the feasibility, usability and stakeholder views of implementing an automated system to monitor the SDM process for surgery in real-time.

Phase 2: Co-develop and refine the intervention with patients and professionals to understand how the intervention works, for whom, and in what context using findings from Phase 1.

Phase 3: Evaluate the effectiveness, cost-effectiveness, and implementation of the intervention to improve patient and health service outcomes in the English NHS.

This protocol describes Phase 1. Details of subsequent phases which will complete the development (Phase 2) and evaluation of the intervention (Phase 3) will be described in future publications.

Methods

For peer review only - http://bmjopen.bmj.com/site/about/guidelines.xhtml

The project will employ mixed-methods to develop a complex intervention comprising multiple components that will impact a wide range of stakeholders and system processes. The overall aim to develop and evaluate the intervention will be conducted according to Medical Research Council (MRC) guidelines [20]. Phase 1 reported here is consistent with the MRC framework's feasibility phase, with consideration of the core elements critical for complex intervention research. Any qualitative elements will be reported in accordance with the consolidated criteria for reporting qualitative studies (COREQ) guidelines [21].

Conceptualisation

There is no unified definition of SDM. A systematic review identified 40 SDM models currently available with 53 different elements clustered in 24 overarching components [1]. Components present in more than half of models were: 'describe treatment options' (88% of models); 'make a decision' (75%); 'patient preferences' (68%); 'tailor information' (65%); 'deliberate' (58%); 'create choice awareness' (55%) and 'learn about the patient' (55%).

This study will conceptualise SDM using the 'Three Talk model' (2012) [22], later refined to 'Implement-SDM' (2019) [23]. This single-component model provides a guide for enhancing health professional communication to deliver SDM, and is the most highly referenced model (>1800 citations, Web of Science). It involves three key steps consistent with other models of SDM: 1) introduction of choice, 2) describing options and 3) helping patients explore preferences and make decisions. This was the chosen model for an NHS MAGIC programme [7] and is recommended in NICE guidelines [24].

Setting

Research will be conducted at three UK hospital trusts (North Bristol Trust/NBT, University Hospitals Bristol and Weston NHS Foundation Trust/UHBWFT, and Bradford Teaching Hospitals NHS Foundation Trust/BTHFT) alongside quality improvement programmes to improve SDM. NBT is one of the largest acute NHS trusts in the UK [25]. It provides a full range of acute clinical care for both local and regional clinical commissioning groups in South-West England. Specialised services are provided through NHS England, Welsh Health Boards and Welsh Specialist Commissioners. Services provided include elective and emergency gastrointestinal surgery, obstetrics and gynaecology, as well as specialist regional services in urology, neurosciences, trauma and orthopaedic and vascular surgery. One UHBWFT department is included as the South-West England regional cardiac surgical

For peer review only - http://bmjopen.bmj.com/site/about/guidelines.xhtml

centre. BTHFT is an acute Trust in the North of England with a full range of elective and emergency surgical services. Bristol and Bradford were purposively selected to maximise reach to ensure a diverse representative sample is included (e.g. 26.8% classed as Asian or Asian British, compared with 5.5% in Bristol).

Phase 1: Assess the feasibility, usability and stakeholder views of implementing an automated system to monitor the SDM process for surgery in real-time

Phase 1 will determine whether it is feasible and acceptable to monitor SDM processes for surgery automatically and in real-time using a novel electronic system. Objectives are to explore:

1.1 Feasibility of automated data collection
1.2 Usability of the electronic measurement system
1.3 Views of diverse stakeholders to inform the use of the system to improve SDM

Each objective comprises separate methods which are described in turn below. This phase is expected to continue until June 2025.

1.1 Feasibility of automated data collection

Feasibility assessment is designed to establish the feasibility of automated real-time evaluation of patient experiences of SDM at scale and will identify opportunities to optimise recruitment and data collection.

Participants

All patients over the age of 18 who have been booked for planned vascular, gastrointestinal, urological, neurosurgical, gynaecological, breast, cardiac and orthopaedic surgical procedures at participating hospitals will be eligible to participate. Surgical departments have been selected to be broadly representative of a diverse range of surgical specialties. Excluded will be patients under the age of 18, those without decisional capacity to consent for medical procedures, or undergoing unplanned (emergency) surgery or endoscopic procedures. Data related to eligibility criteria are routinely collected through electronic patient record (EPR) systems.

For peer review only - http://bmjopen.bmj.com/site/about/guidelines.xhtml

Measurement of patient experience

Real-time measurement of patient experiences of the process of SDM will be facilitated by a secure, automated system procured through a third-party provider approved by NHS trusts. The system is a customisable off-the-shelf electronic patient-reported outcome measurement software and has previously been used for electronic data capture in other countries. Eligible patients will be identified through EPR using algorithms developed in collaboration with the software provider. Structured data queries will be designed to extract details of patients booked for eligible procedures. Queries will be designed to run automatically, securely transferring data from the hospital to the software provider daily to account for changes in scheduling. The automated system will send three validated SDM measurement instruments within one day after surgery booking (real-time baseline measurement). This timepoint in the decision making process was chosen as a pragmatic point in time to represent patients' cumulative experiences of SDM for surgery which may include discussions with surgeons, physicians, general practitioners, nurses, family, and friends. The selected measurement instruments will be operationalised into an online survey and administered via short messaging service (SMS) or email. A reciprocal data feed will securely return patients' survey responses immediately to the hospital data warehouse for secure storage (real-time analysis and feedback). Follow-up measures are sent within one day before surgery by either SMS or email (real-time follow-up measurement). A schematic of the process and intervention aims is illustrated in Figure 1.

Selection of three SDM measures was made through discussions within the study team and were informed by a systematic review of SDM measurement instruments using COSMIN (consensus-based standards for the selection of health measurement instruments) methods [26], national guidelines [19] and recommendations and use within NHS clinical practice [27–29]. The CollaboRATE instrument is a validated 3-item patient-reported measure assessing the extent of SDM experienced by patients [30]. Assessment of the instrument using COSMIN methods demonstrated acceptable discriminative validity, concurrent validity, intra-rater reliability and sensitivity to change [26]. It has been used in excess of 40 studies [31], including evaluations of quality improvement projects in surgery [32]. The SDM-Q-9 instrument is a validated 9-item patient-reported measure that evaluates their perceptions of involvement in the decision-making process [33]. It has been widely used in interventional studies and demonstrates good reliability, structural validity [26,33]. Systematic review evidence recommended use of SDM-Q-9 for surgery [34]. The SHARED-Q10 instrument is a 10-item patient-reported measure to assess patient perceptions of information provided, involvement in consultations, and agreement with the decision made [35]. This measure is included because it was developed, validated, and used in an NHS quality improvement programme [36,37]

For peer review only - http://bmjopen.bmj.com/site/about/guidelines.xhtml

and evaluates domains beyond patient perception of professional communication. Complete measurement instruments can be found in Supplemental File, Figures S1_S3.

Analysis

Feasibility of real-time monitoring will be evaluated by analysis of overall recruitment rate, response rates and time to response for the SDM measures at baseline and follow-up. Response rates will be presented as a number and percentage based on patients who completed the measures (e.g. completed all three items of the CollaboRATE instrument). Issues of equality, diversity and inclusion will be explored by examining the correlation between responders/non-responders and sociodemographic patient variables extracted from EPR.

Relationships between responders and non-responders and clinical and sociodemographic details will be explored using uni- and multivariable logistic regression. Included will be age, sex, ethnicity, index of multiple deprivation, and clinical and treatment parameters (e.g. operation (three-digit OPCS code), diagnosis (ICD10), date of booking, specialty, number of outpatient appointments in relevant specialty, number and length of hospital inpatient episode in relevant specialty). Index of multiple deprivation will be derived by Lower Layer Super Output Area (LSOAs) for individuals' postcode. All variables will be extracted from routinely collected data in EPR.

In addition, the study team will document any relevant technical, financial, administrative, and logistical observations throughout the study and pertinent challenges using shared electronic records (e.g. Microsoft Office suite). Any learning points will be descriptively summarised.

1.2 Usability of the electronic measurement system

Post-deployment usability testing will be conducted according to International Standards Organisation (ISO) standards for human-systems interaction (9241-11:2018) to evaluate the system's use in this context [38,39]. System users, defined as anyone who is a current or prospective surgical patient, will be invited to participate in a mixed-method usability evaluation to assess system effectiveness, system efficiency and user satisfaction (see Box 1). To complete usability evaluation, a process map will be created to define the number and type of task required to complete the measurement system.

Box 1 Definition of usability concepts

For peer review only - http://bmjopen.bmj.com/site/about/guidelines.xhtml

System effectiveness: the ability of participants to perform tasks to achieve pre-determined goals completely and accurately, and without negative consequences (e.g. poor layout of the system interface leading to participants missing or accidentally selecting system options) [38–41].

System efficiency: the amount of participant resources required to achieve the pre-specified goals (e.g. system completion time) [41,42].

User satisfaction: the subjective opinions of participants based on their experience interacting with the system [41]. This includes any subjective reports about likes, dislikes and recommendations for changes [38].

System effectiveness

One-to-one user testing sessions will be used to assess system effectiveness by evaluating task completion and error rates. Sessions will involve completing the automated system in a simulated environment, applying concurrent think aloud techniques [43–45]. A topic guide will be developed and will structure the testing session discussions (Supplemental File 1, Table S1).

Patient and public representatives will be invited to participate in online user testing sessions. They will be eligible if they are over the age of 18. Individuals from two patient experience panels (NBT, BTHFT) will be recruited through respective panel coordinators. Sampling will be purposive to maximise variation in geographical location, ethnicity, and sex and will aim to include individuals whose first language is not English.

User testing will be completed using a video-conferencing software (e.g. Zoom, MS Teams) and audio-recorded. Two researchers familiar with the automated system and trained in qualitative research will conduct the user testing sessions. Observational notes will be taken to collect further information about challenges or errors encountered during task completion [46,47].

Task completion rates will be calculated as percentage of tasks completed. Error rates will be calculated based on number of user errors encountered. User errors will be deviations or problems experienced that will interfere with successfully completing the task. Number and type of non-critical errors (successfully addressed by testers themselves following instructions from the observer) and critical errors (require the observer to intervene or take remedial actions) will be noted. Results will be presented using descriptive statistics.

Understanding of system effectiveness will be supplemented by analysis of response rates generated through feasibility work in 1.1.

For peer review only - http://bmjopen.bmj.com/site/about/guidelines.xhtml

For peer review only

System efficiency

System efficiency will be assessed by calculating task completion time and task efficiency. Task completion time is defined as the time participants took from the first activity (starting the survey by following the hyperlink) to the last activity (submission of the survey). Task efficiency is defined as the time spent to complete each task. Analyses will be based on those who completed the automated system and for whom first and last activity timestamps were available.

User satisfaction

One-to-one user interviews will be conducted to assess user satisfaction in-depth. Interviews will explore issues including ease of use/navigation, satisfaction with instructions, satisfaction with the visual display, ease of access, burden, and likelihood of using the system again. Barriers and facilitators to completing the measurement system will also be explored. A topic guide will be tested and refined and used to direct discussions.

A sub-set of eligible patients and participants of the user testing sessions will be invited to take part. A purposive sampling strategy will be adopted to ensure that insights are drawn from a range of perspectives. Sampling characteristics will be 1) experience with surgery (vascular, gastrointestinal, urological, neurosurgical, gynaecological, breast or orthopaedic surgery) and good/bad SDM experience, 2) sex, 3) age, 4) ethnicity and 5) individuals whose first language is not English. Participant characteristics will be assessed as the study progresses and recruitment efforts will focus to target under-represented patients as necessary. Recruitment of the subset of patient participants will be undertaken by the principal investigator, research nurse or clinical collaborators via email or telephone. User testing participants will be recruited by researchers during the user testing sessions and interviews will be conducted immediately following the user testing session.

Interviews will be conducted primarily remotely (e.g. telephone or video conference) by experienced and trained qualitative researchers. All audio-recorded interviews will be transcribed and anonymised. Transcripts will be thematically analysed (see Qualitative analysis section in 1.3).

1.3 Views of diverse stakeholders to inform the use of the system to improve SDM

Qualitative research with a wide range of stakeholders (including patients, healthcare professionals and members of the community) will be conducted to understand views of multiple stakeholders to

For peer review only - http://bmjopen.bmj.com/site/about/guidelines.xhtml

inform the use of the system to improve SDM. Opinions about the acceptability and potential impact of real-time monitoring of SDM will be sought. Views on potential intervention components (activities), mechanisms of change, intermediate outcomes, assumptions, and indicators will be explored. Results will be used to co-develop initial programme theory to inform Phase 2.

Patients and members of the public and community over the age of 18 will be eligible to take part. The sample will include people who are disproportionately affected by a poor SDM process and outcomes of surgery: those that are economically disadvantaged, from minority ethnic groups, and in older age [48–51]. Professionals working in participating Trusts will be eligible for inclusion and may include surgeons, anaesthetists, nurses, perioperative care physicians, allied health professionals and hospital managers.

Recruitment

Eligible participants will be identified through existing networks, collaborations with local hospital patient panels, community leaders, and patients who have participated in feasibility (1.1) and usability (1.2) data collection. We will seek to recruit individuals who experience multiple intersecting inequalities to ensure the views of those with barriers to accessing healthcare are incorporated [48]. Recruitment of members of the community will be conducted using techniques developed and successfully applied by the Born in Bradford team [52,53] and the patient and public involvement and engagement (PPIE) group of the NIHR Bristol BRC. Recruitment materials will be translated into most spoken languages within the local areas.

Purposive sampling will seek to achieve diversity in relation to socio-demographic characteristics (e.g. age, gender), experience with surgery or SDM (e.g. surgical specialty, good/bad SDM experience) or under-served groups (economically disadvantaged, older age, ethnic minority). Where appropriate, snowball sampling will also be used, whereby individuals who participate in the study are asked about other potentially interested participants. The sample size will ultimately depend on theoretical saturation (e.g. when no new insights are identified from the data and sufficient data are collected to address the research question) [54,55]. It is anticipated that approximately 130 participants (around 105 patients and members of the community, and 25 professionals) will be required.

Data collection

Data collection will apply a flexible strategy to minimise perception that the research is 'hard-to-engage-with' [52]. A range of qualitative research methods are planned remotely and/or face-to-

For peer review only - http://bmjopen.bmj.com/site/about/guidelines.xhtml

face including semi-structured interviews, focus groups and participatory approaches (e.g. community events, discussion groups). It is anticipated that a minimum of 30 one-to-one interviews and six focus groups are required, complemented by recruitment through community events and discussion groups. However, these methods may be adapted based on evolving best-practice evidence from citizen science [56] and feedback from PPIE stakeholders. For example, evidence suggests that some British Asian people may be more willing to participate in a focus group in a familiar setting (e.g. community centre) than other settings [57].

Interviews and focus groups will be facilitated by experienced qualitative researchers based in Bristol and Bradford. Topic guides for interviews and focus groups will be developed to direct discussions. This will be iteratively refined during data collection to explore emergent views. Interviews and focus groups will be held face-to-face, over the telephone or using a secure video conference service (e.g. Zoom MS, Teams,) but will ultimately depend on participant preference. Data collection will primarily be conducted in English. However, where data are collected from non-English speaking members of the community, additional support will be provided by interpreters and specialist researchers who conduct relevant foreign language interviews and focus groups. All interviews and focus groups will be audio recorded and transcribed verbatim. Field notes will be taken during the interviews.

Qualitative analyses

Transcripts and field notes will be analysed using a thematic approach with the help of a qualitive data management software (NVivo). Principles of thematic analysis will be applied to the data whereby 1) transcripts and notes will be read and re-read, 2)codes are generated and assigned to relevant excerpts within the transcripts, 3) themes will be identified by collating similar codes, 4) accuracy of themes will be checked and 5) detailed analysis of themes will take place [58]. Analysis will involve linking transcripts and observational notes by integrating relevant data from both sources to gain a more comprehensive understanding of key findings. This process will primarily be inductive, with codes developed and iteratively refined through interpretation of the data. There will, however, be an a priori interest in examining data in relation to the study aims. For example, information to support evidence for the acceptability of monitoring the SDM process, and impact of monitoring on clinical care will actively be sought.

Analyses will be conducted separately for different stakeholder groups (patients, professionals, community) to help ascertain different viewpoints or experiences reported by each participant

For peer review only - http://bmjopen.bmj.com/site/about/guidelines.xhtml

group. Depending on findings, an additional layer of analysis may be conducted to contrast results for several sub-groups (e.g. different under-served groups; different specialties) to ensure differing perspectives and experiences by population and context are accounted for in later intervention development. At least two experienced qualitative researchers will perform analysis independently and meet regularly to discuss impressions of the data. A subset of transcripts will be double coded by another experienced qualitative researcher. Any discrepancies in coding or interpretation of data will be referred to the wider study team for further discussions.

Summaries of findings from the analyses (descriptive reports) will be written, combining preliminary findings from the various data sources in relation to the study objectives. Drafts of these summaries will be prepared following rounds of recruitment and analyses and discussed within the study team. The summaries will be iteratively developed as analysis proceeds and will inform discussions about saturation.

Dedicated multi-disciplinary meetings involving public contributors will be held to articulate an initial programme theory to inform the future development of the intervention to be more inclusive of recognised under-served groups. A summary of key findings from qualitative data collection in 1.3. will be prepared. We will draw on behavioural (COM-B) [59] and organisational (Normalisation Process Theory) [60] change theory to identify theory of how the intervention will work for under-served groups. Summaries will be combined to form a comprehensive report, providing a basis for Phase 2.

Data management

All data will be generated and handled in accordance with relevant directives and regulations (e.g. Data Protection Act 2018). Any data collected as part of qualitative data collection will be recorded using encrypted devices. Audio files will be securely transferred and transcribed by transcription services approved by the University of Bristol. Transcripts will ensure anonymity of participants (e.g. in future study outputs) by assigning pseudonyms or participant IDs to replace any names or identifiable information. All electronic data files will be saved in restricted folders only accessible to the research team, on secure University of Bristol network space that adheres to the University of Bristol's data security policies. Files containing any personal information (e.g. contact details) will exclusively use the linked participant ID and will be encrypted and stored securely on the university servers.

For peer review only - http://bmjopen.bmj.com/site/about/guidelines.xhtml

For peer review only

Study steering group

A dedicated study steering group will be convened to provide oversight and strategic direction for the study. It will include patients and independent clinical and methodological experts and will meet six monthly to review progress and provide strategic guidance.

Patient and public involvement and engagement

PPIE is central to the project and will play a key role throughout. Patient partners have helped define the research questions and draft the protocol. A PPIE strategy has been developed in collaboration with patient partners in the planning stages of this study to ensure it meets the needs of patients. It includes PPIE activities across 1) strategy and oversight, 2) study conduct and 3) dissemination. Involvement of the patient co-author (VS), a patient advisory group consisting of members from a diverse background and patient representatives on our steering group will ensure the study focuses on patient needs throughout. PPIE activities will be co-ordinated by an experienced researcher and will be evaluated. Any feedback will be used to iteratively evolve the PPIE strategy to meet the needs of advancing PPIE practices.

Ethics and dissemination

This study is part of a project spanning quality improvement and research. It is therefore subject to two governance processes requiring separate approvals: Approval to monitor patients' experience of SDM in routine clinical practice was initially approved through a quality improvement proposal at North Bristol NHS Trust (reference: Q80008). This was then incorporated into a larger programme of work, where all processes were approved through the appropriate governance framework (Consent & SDM Programme Board, reporting to an Executive Assurance Committee). Patients will provide consent to participate in real-time monitoring through indicating their agreement with Terms and Conditions for the programme of work before completing the survey administered through the measurement system.

Ethical approval required to conduct qualitative data collection with NHS patients and professionals was granted by the NHS HRA North West - Liverpool Central Research Ethics Committee (reference:

For peer review only - http://bmjopen.bmj.com/site/about/guidelines.xhtml

21/PR/0345). Participants will provide written consent to participate in qualitative data collection before any research activity will commence. Consent will be obtained electronically through a link to a secure data management platform (RedCap Version 11.1.18). As part of the consent process, participants will agree to their anonymised quotes being published in scientific journals.

The results of this work will be presented to professionals (at conferences, as journal articles), shared with the public (social media, engagement events) and those who participated in the project. We will collaborate with organisations involved in SDM (National Institute for Health and Care Excellence, NHS England) to share findings from the study and maximise the value of our work. Materials produced for dissemination will be tailored to the target audience and will include plain summaries in various languages, formal and informal presentations, infographics, or posters.

Author contributions

AM developed the original idea for this study along with KNLA and JMB. AM, KNLA, CH, RCM, JMB, DH, SH, CC, JH, BG, LR, AW, JA, HB contributed to the development of the research question and objectives and were involved in the design of the study protocol. KNLA, RCM, CC, LR, JH, HB provide methodological expertise. CH and AM wrote the first draft of the manuscript, and all co-authors reviewed and critically appraised the manuscript. AM (guarantor) has overall responsibility for the content and project with strategic oversight from JMB. VS contributed to the PPIE strategy. Collaborators part of the ALPACA Study team (AJ, AS, AL, BR, JP, MRW, PC, PB, SP, TB, TW) provide clinical liaison and subject expertise that have shaped the study design. All collaborators have critically reviewed the study proposal. All authors read and approved the final version.

Funding statement

This project is funded by the National Institute for Health and Care Research (NIHR) Programme Development Grant (Co-Chief Investigators: AM & JB). It is also supported by an NIHR Clinician Scientist award to AM (NIHR CS-2017-17-010) and by the NIHR Biomedical Research Centre (BRC) at the University Hospitals Bristol and Weston NHS Foundation Trust and the University of Bristol (BRC-1215-20011). The views expressed in this publication are those of the authors and not necessarily reflect those of the NIHR, the Department of Health and Social Care or the UK National Health Service.

For peer review only - http://bmjopen.bmj.com/site/about/guidelines.xhtml

Competing interests

All authors declare no competing interests.

Data availability statement

Relevant anonymised data will be included in published manuscripts and/or made available as supplementary files. Participant level data and the datasets generated and/or analysed during the current project will be stored in a non-publicly available repository. Pseudo-anonymised datasets will be made available via the University of Bristol's Research Data Repository, data.bris to bona fide researchers, subject to a legally binding data access agreement. Any applications to access data will involve a case-by-case review by the University of Bristol Data Access Committee. Qualifying researchers will be required to sign a data access agreement and closely liaise with study team members to ensure that the data they plan to make public are sufficiently anonymised. Generally, data will be made available for non-commercial use, only for the purpose of health and care research and with appropriate approvals in place.

For peer review only

REFERENCES

1 Bomhof-Roordink H, Gärtner FR, Stiggelbout AM, *et al.* Key components of shared decision making models: A systematic review. BMJ Open. 2019;**9**. doi:10.1136/bmjopen-2019-031763

2 Department of Health and Social Care. The NHS Constitution for England. 2021.https://www.gov.uk/government/publications/the-nhs-constitution-for-england/the-nhs-constitution-for-england#introduction-to-the-nhs-constitution (accessed 15 Sep 2022).

3 National Health Service. The NHS Long Term Plan. Published Online First: 2019.www.longtermplan.nhs.uk (accessed 15 Sep 2022).

4 Archive BLUW. Health and Social Care Act 2012 - NHS Reforms. 2013.https://www.webarchive.org.uk/en/ukwa/collection/9 (accessed 11 May 2023).

5 Royal College of Surgeons. Good Surgical Practice. 2013. https://www.rcseng.ac.uk/standards-and-research/gsp/ (accessed 15 Sep 2022).

6 General Medical Council. Decision making and consent - ethical guidance. 2020. https://www.gmc-uk.org/ethical-guidance/ethical-guidance-for-doctors/decision-making-and-consent (accessed 15 Sep 2022).

7 Joseph-Williams N, Lloyd A, Edwards A, *et al.* Implementing shared decision making in the NHS: lessons from the MAGIC programme. *BMJ* 2017;**357**:j1744. doi:10.1136/bmj.j1744

8 Lagarde SM, Franssen SJ, Van Werven JR, *et al.* Patient preferences for the disclosure of prognosis after esophagectomy for cancer with curative intent. *Ann Surg Oncol* 2008;**15**:3289–98. doi:10.1245/s10434-008-0068-y

9 Légaré F, Adekpedjou R, Stacey D, *et al.* Interventions for increasing the use of shared decision making by healthcare professionals. *Cochrane database Syst Rev* 2018;**7**:CD006732. doi:10.1002/14651858.CD006732.PUB4

10 Snijders HS, Kunneman M, Bonsing BA, *et al.* Preoperative risk information and patient involvement in surgical treatment for rectal and sigmoid cancer. *Color Dis* 2014;**16**:O43–9. doi:10.1111/CODI.12481

11 Abbott TEF, Fowler AJ, Dobbs TD, *et al.* Frequency of surgical treatment and related hospital procedures in the UK: a national ecological study using hospital episode statistics. *BJA Br J Anaesth* 2017;**119**:249–57. doi:10.1093/BJA/AEX137

12 Care Quality Commission. Better care in my hands. 2016. https://www.cqc.org.uk/sites/default/files/20160519_Better_care_in_my_hands_FINAL.pdf%0Ahttp://www.cqc.org.uk/content/better-care-my-hands-review-how-people-are-involved-their-care

13 Elwyn G, Frosch DL, Kobrin S. Implementing shared decision-making: Consider all the consequences. *Implement Sci* 2016;**11**:1–10. doi:10.1186/S13012-016-0480-9/TABLES/2

14 Hughes TM, Merath K, Chen Q, *et al.* Association of shared decision-making on patient-reported health outcomes and healthcare utilization. *Am J Surg* 2018;**216**:7–12. doi:10.1016/J.AMJSURG.2018.01.011

15 Arterburn D, Wellman R, Westbrook E, *et al.* Introducing decision aids at group health was linked to sharply lower hip and knee surgery rates and costs. *Health Aff* 2012;**31**:2094–104. doi:10.1377/hlthaff.2011.0686

For peer review only - http://bmjopen.bmj.com/site/about/guidelines.xhtml

16 Kirsch M, Brown S, Smith BW, *et al.* The Presence and Persistence of Unrealistic Expectations in Patients Undergoing Nerve Surgery. *Neurosurgery* 2020;**86**:778–82. doi:10.1093/NEUROS/NYZ335

17 Homer CV, Tod AM, Thompson AR, *et al.* Expectations and patients' experiences of obesity prior to bariatric surgery: a qualitative study. *BMJ Open* 2016;**6**:e009389. doi:10.1136/BMJOPEN-2015-009389

18 Heisler M, Bouknight RR, Hayward RA, *et al.* The Relative Importance of Physician Communication, Participatory Decision Making, and Patient Understanding in Diabetes Self-management. *J Gen Intern Med* 2002;**17**:243–52. doi:10.1046/J.1525-1497.2002.10905.X

19 National Institute for Health and Care Excellence. Shard decision making [NG197]. Evidence reviews 2021. NICE 2021. https://www.nice.org.uk/guidance/ng197/evidence/b-interventions-to-support-effective-shared-decision-making-pdf-9142344255 (accessed 15 Sep 2022).

20 Skivington K, Matthews L, Simpson SA, *et al.* A new framework for developing and evaluating complex interventions: update of Medical Research Council guidance. *BMJ* 2021;**374**:n2061. doi:10.1136/BMJ.N2061

21 Tong A, Sainsbury P, Craig J. Consolidated criteria for reporting qualitative research (COREQ): A 32-item checklist for interviews and focus groups. *Int J Qual Heal Care* 2007;**19**:349–57. doi:10.1093/intqhc/mzm042

22 Elwyn G, Frosch D, Thomson R, *et al.* Shared decision making: A model for clinical practice. J. Gen. Intern. Med. 2012;**27**:1361–7. doi:10.1007/s11606-012-2077-6

23 Joseph-Williams N, Williams D, Wood F, *et al.* A descriptive model of shared decision making derived from routine implementation in clinical practice ('Implement-SDM'): Qualitative study. *Patient Educ Couns* 2019;**102**:1774–85. doi:10.1016/j.pec.2019.07.016

24 National Institute for Health and Care Excellence. Shared decision making (NG197). 2021.

25 Care Quality Commission. North Bristol NHS Trust Inspection Report. 2019.

26 Gärtner FR, Bomhof-Roordink H, Smith IP, *et al.* The quality of instruments to assess the process of shared decision making: A systematic review. *PLoS One* 2018;**13**. doi:10.1371/journal.pone.0191747

27 NHS England and NHS Improvement. Personalised Care Shared Decision Making Summary guide. 2019. https://www.england.nhs.uk/wp-content/uploads/2019/01/shared-decision-making-summary-guide-v1.pdf (accessed 26 Jan 2023).

28 NHS England Personalised Care. Shared Decision Making - Summary guide. https://www.england.nhs.uk/shared-decision-making/guidance-and-resources/ (accessed 15 Sep 2022).

29 Barr PJ, Forcino RC, Thompson R, *et al.* Evaluating CollaboRATE in a clinical setting: analysis of mode effects on scores, response rates and costs of data collection. *BMJ Open* 2017;**7**:e014681. doi:10.1136/BMJOPEN-2016-014681

30 Elwyn G, Barr PJ, Grande SW, *et al.* Developing CollaboRATE: A fast and frugal patient-reported measure of shared decision making in clinical encounters. *Patient Educ Couns* 2013;**93**:102–7. doi:10.1016/J.PEC.2013.05.009

31 Scholl I, Loon MK Van, Sepucha K, *et al.* Measurement of shared decision making – a review of instruments. *Z Evid Fortbild Qual Gesundhwes* 2011;**105**:313–24.

For peer review only - http://bmjopen.bmj.com/site/about/guidelines.xhtml

doi:10.1016/J.ZEFQ.2011.04.012

32 Brodney S, Fowler FJ, Barry MJ, *et al.* Comparison of Three Measures of Shared Decision-Making: SDM Process_4, CollaboRATE, and SURE Scales. *Med Decis Making* 2019;**39**:673. doi:10.1177/0272989X19855951

33 Kriston L, Scholl I, Hölzel L, *et al.* The 9-item Shared Decision Making Questionnaire (SDM-Q-9). Development and psychometric properties in a primary care sample. *Patient Educ Couns* 2010;**80**:94–9. doi:10.1016/J.PEC.2009.09.034

34 de Mik SML, Stubenrouch FE, Balm R, *et al.* Systematic review of shared decision-making in surgery. *Br J Surg* 2018;**105**:1721–30. doi:10.1002/bjs.11009

35 Durand MA, Bekker HL, Casula A, *et al.* Can we routinely measure patient involvement in treatment decision-making in chronic kidney care? A service evaluation in 27 renal units in the UK. *Clin Kidney J* 2016;**9**:252–9. doi:10.1093/CKJ/SFW003

36 NHS Rightcare. Measuring Shared Decision Making: A review of research evidence - A report for the Shared Decision Making programme in partnership with Capita Group Plc. London: 2012. https://www.england.nhs.uk/wp-content/uploads/2013/08/7sdm-report.pdf

37 NHS Rightcare. Your Health – Your Decision. London: 2013. https://aqua.nhs.uk/wp-content/uploads/2021/10/Your-Health-Your-Decision-Evaluation-Report.pdf

38 Bevan N, Carter J, Earthy J, *et al.* New ISO Standards for Usability, Usability Reports and Usability Measures. *Lect Notes Comput Sci (including Subser Lect Notes Artif Intell Lect Notes Bioinformatics)* 2016;**9731**:268–78. doi:10.1007/978-3-319-39510-4_25

39 ISO. ISO 9241-11:2018(en), Ergonomics of human-system interaction — Part 11: Usability: Definitions and concepts. 2018;:9241(11).https://www.iso.org/obp/ui/#iso:std:iso:9241:-11:ed-2:v1:en (accessed 24 Nov 2021).

40 Kyte D, Anderson N, Auti R, *et al.* Development of an electronic patient-reported outcome measure (ePROM) system to aid the management of patients with advanced chronic kidney disease. *J Patient-Reported Outcomes* 2020;**4**. doi:10.1186/s41687-020-00223-8

41 Aiyegbusi OL. Key methodological considerations for usability testing of electronic patient-reported outcome (ePRO) systems. *Qual Life Res* 2020;**29**:325–33. doi:10.1007/s11136-019-02329-z

42 Barnum CM. Establishing the essentials. In: Barnum CM, ed. *Usability testing essentials*. Boston: : Morgan Kaufmann 2011. 9–23.https://scholar.google.com/scholar?hl=en&as_sdt=0%2C5&q=Barnum%2C+C.+M.+%282011%29.+Establishing+the+essentials&btnG= (accessed 27 Jan 2023).

43 van den Haak MJ, de Jong MDT, Schellens PJ. Evaluation of an Informational Web Site: Three Variants of the Think-aloud Method Compared. *Tech Commun* 2007;**54**:58–71.

44 Alshammari T, Alhadreti O, Mayhew P. When to ask participants to think aloud:A comparative study of concurrent and retrospective think-aloud methods. *Int J Hum Comput Interact* 2015;**6**:48–64.

45 Khajouei R, Farahani F. A combination of two methods for evaluating the usability of a hospital information system. *BMC Med Inform Decis Mak* 2020;**20**:1–10. doi:10.1186/S12911-020-1083-6/TABLES/4

46 Farzanfar R, Finkelstein J, Friedman RH. Testing the Usability of Two Automated Home-Based Patient-Management Systems. *J Med Syst 2004 282* 2004;**28**:143–53.

For peer review only - http://bmjopen.bmj.com/site/about/guidelines.xhtml

doi:10.1023/B:JOMS.0000023297.50379.3C

47 Kaufman DR, Patel VL, Hilliman C, *et al.* Usability in the real world: assessing medical information technologies in patients' homes. *J Biomed Inform* 2003;**36**:45–60. doi:10.1016/S1532-0464(03)00056-X

48 Chen JC, Obeng-Gyasi S. Intersectionality and the Surgical Patient: Expanding the Surgical Disparities Framework. *Ann Surg* 2022;**275**:E3–5. doi:10.1097/SLA.0000000000005045

49 Durand MA, Carpenter L, Dolan H, *et al.* Do interventions designed to support shared decision-making reduce health inequalities? A systematic review and meta-analysis. *PLoS One* 2014;**9**. doi:10.1371/JOURNAL.PONE.0094670

50 Tarver WL, Haggstrom DA. The Use of Cancer-Specific Patient-Centered Technologies Among Underserved Populations in the United States: Systematic Review. *J Med Internet Res* 2019;**21**:e10256. doi:10.2196/10256

51 De Acedo Lizárraga MLS, De Acedo Baquedano MTS, Cardelle-Elawar M. Factors that affect decision making: Gender and age differences. *Int J Psychol Psychol Ther* 2007;**7**:381–91.https://www.redalyc.org/pdf/560/56070306.pdf (accessed 1 Nov 2023).

52 Islam S, Joseph O, Chaudry A, *et al.* "We are not hard to reach, but we may find it hard to trust" …. Involving and engaging 'seldom listened to' community voices in clinical translational health research: a social innovation approach. *Res Involv Engagem 2021 71* 2021;**7**:1–15. doi:10.1186/S40900-021-00292-Z

53 Dickerson J, Bird PK, Bryant M, *et al.* Integrating research and system-wide practice in public health: Lessons learnt from Better Start Bradford. *BMC Public Health* 2019;**19**:1–12. doi:10.1186/S12889-019-6554-2/TABLES/8

54 Saunders B, Sim J, Kingstone T, *et al.* Saturation in qualitative research: exploring its conceptualization and operationalization. *Qual Quant* 2018;**52**:1893. doi:10.1007/S11135-017-0574-8

55 Hennink MM, Kaiser BN, Marconi VC. Code Saturation Versus Meaning Saturation: How Many Interviews Are Enough? *Qual Health Res* 2017;**27**:591–608. doi:10.1177/1049732316665344

56 Vohland K, Land-Zandstra A, Ceccaroni L, *et al. The Science of Citizen Science*. Cham: : Springer 2021. doi:10.1007/978-3-030-58278-4

57 Humm C, Schrögel P. Science for All? Practical Recommendations on Reaching Underserved Audiences. *Front Commun* 2020;**5**:42. doi:10.3389/FCOMM.2020.00042/BIBTEX

58 Braun V, Clarke V. *Successful qualitative research : a practical guide for beginners*. SAGE 2013.

59 Michie S, van Stralen MM, West R. The behaviour change wheel: A new method for characterising and designing behaviour change interventions. *Implement Sci* 2011;**6**:1–12. doi:10.1186/1748-5908-6-42/TABLES/3

60 Murray E, Treweek S, Pope C, *et al.* Normalisation process theory: A framework for developing, evaluating and implementing complex interventions. *BMC Med* 2010;**8**:1–11. doi:10.1186/1741-7015-8-63/TABLES/3

For peer review only - http://bmjopen.bmj.com/site/about/guidelines.xhtml

Figures

Figure 1. Schematic of measurement process

For peer review only

For peer review only - http://bmjopen.bmj.com/site/about/guidelines.xhtml

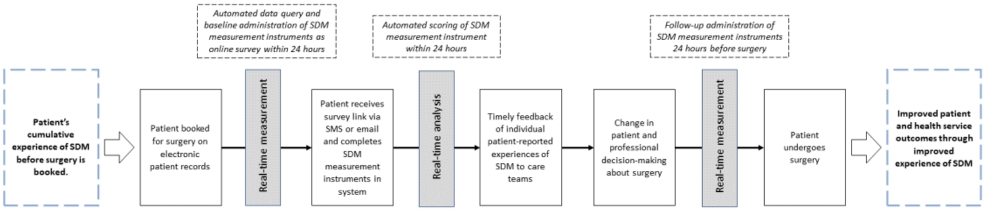

Figure 1. Schematic of measurement process

1086x378mm (38 x 38 DPI)

Supplemental File 1: The ALPACA Study

1. Instruments to measure the process of shared decision making

Figure S1 CollaboRATE measure

collaboRATE™

1. How much effort was made to help you understand your health issues?

0	1	2	3	4	5	6	7	8	9
No effort was made									Every effort was made

2. How much effort was made to listen to the things that matter most to you about your health issues?

0	1	2	3	4	5	6	7	8	9
No effort was made									Every effort was made

3. How much effort was made to include what matters most to you in choosing what to do next?

0	1	2	3	4	5	6	7	8	9
No effort was made									Every effort was made

From: Elwyn, Glyn, Paul James Barr, Stuart W. Grande, Rachel Thompson, Thom Walsh, and Elissa M. Ozanne. "Developing CollaboRATE: a fast and frugal patient-reported measure of shared decision making in clinical encounters." *Patient education and counseling* 93, no. 1 (2013): 102-107.

Figure S2 The 9-item Shared Decision Making Questionnaire (SDM-Q-9)

The 9-item Shared Decision Making Questionnaire (SDM-Q-9)

[Example] **Please indicate which health complaint/problem/illness the consultation was about:**

[Example] **Please indicate which decision was made:**

Nine statements related to the decision-making in your consultation are listed below. For each statement please indicate how much you agree or disagree.

	completely disagree	strongly disagree	somewhat disagree	somewhat agree	strongly agree	completely agree
**1. My doctor made clear that a decision needs to be made.**	☐	☐	☐	☐	☐	☐
**2. My doctor wanted to know exactly how I want to be involved in making the decision.**	☐	☐	☐	☐	☐	☐
**3. My doctor told me that there are different options for treating my medical condition.**	☐	☐	☐	☐	☐	☐
**4. My doctor precisely explained the advantages and disadvantages of the treatment options.**	☐	☐	☐	☐	☐	☐
**5. My doctor helped me understand all the information.**	☐	☐	☐	☐	☐	☐
**6. My doctor asked me which treatment option I prefer.**	☐	☐	☐	☐	☐	☐
**7. My doctor and I thoroughly weighed the different treatment options.**	☐	☐	☐	☐	☐	☐
**8. My doctor and I selected a treatment option together.**	☐	☐	☐	☐	☐	☐
**9. My doctor and I reached an agreement on how to proceed.**	☐	☐	☐	☐	☐	☐

From: Kriston, L., Scholl, I., Hölzel, L., Simon, D., Loh, A., & Härter, M. (2010). The 9-item Shared Decision Making Questionnaire (SDM-Q-9). Development and psychometric properties in a primary care sample. *Patient education and counseling*, *80*(1), 94-99.

Figure S3 SHARED measure

SHARED – A Patient Experience of Shared Decision Making Questionnaire

What was your experience of making this treatment decision with your [health professional/s] – *for each statement, tick the box that matches best how much you agree with it:*

		Disagree Strongly	Disagree	Not Sure	Agree	Agree Strongly
	The [health] professional…					
**1**	talked about other options from the one I chose	☐	☐	☐	☐	☐
**2**	talked about whether or not there was a medically best option for my *[health problem]*	☐	☐	☐	☐	☐
**3**	gave their views about the options	☐	☐	☐	☐	☐
**4**	asked for my views about the options	☐	☐	☐	☐	☐
	I talked about…					
**5**	what was important to me about this decision	☐	☐	☐	☐	☐
**6**	why one option suited me better than another	☐	☐	☐	☐	☐
**7**	the risks and benefits of the options for me and my health	☐	☐	☐	☐	☐
	I felt…					
**8**	it would be OK to choose any option we talked about	☐	☐	☐	☐	☐
**9**	the decision made was the best one for me	☐	☐	☐	☐	☐
**10**	the *[health]* professional and I agreed which option was the best one for me	☐	☐	☐	☐	☐

From: Bekker HL, Légaré F, Nye A, Walker W. SHARED – A Patient Experience of Shared Decision Making Questionnaire. (2012). University of Leeds, UK

For peer review only

2. Example topic guides for qualitative data collection

Table S1 Usability testing (1.2) – interview guide

Intro

- Thank you and introduction
- Explanation of project (assess to what extent), ask if there are questions
- Ask for preferred mode for completing the survey (sms/email) and send the link
- Check if ok to audio record → *switch on recording*
- Explain purpose of the session:
  - We would like to test the survey that patients receive when booked in for surgery.
  - The focus is on **functionality**. It helps us make improvements to the process.
  - This session is **NOT** about the wording of questions, we are just interested in the usability
  - The text/email is a tester only, so the responses you give are not real
- Explain specific tasks:
  - There are two surveys. 8 steps (3 questions) for the first one, 20 steps (9 questions) for the second one.
  - We will
    - run through these steps and see how you get on with these
    - might feel a little unnatural but is important you tell me what you think and what you see, what is clear/unclear, what is easy/not straight forward or difficult to complete
  - Say where there is a problem, e.g. that you had to press twice to proceed
- Explain there will be questions at the end

Think-aloud exercise

Start with 8 steps of CollaboRATE

- Prompts if participant doesn't talk
  - Can you tell me what you currently see?
  - What are you going to do next?
  - What can you see now?
- Prompts to elicit views
  - Could you tell me what you think about Step X?
  - How do you feel about Step X?
  - What do you think about Step X?
  - How clear is Step X?
  - How easy is Step X?

Pause and ask follow-up questions

- "Having just completed the survey…"
  - How easy do you think is it to respond to the survey?
  - What do you think about the length of the survey?
  - What are your thoughts on the overall visual display? How visually appealing is the survey?
  - What would stop people from doing the survey? *Why?*
  - What issues can you think of people might encounter when completing the survey? *Why?*
  - What else would you change about how the survey is delivered? *Why?*

For peer review only

Table S2 Exploring views of under-served groups (1.3) – interview guide

Background

- Intro to interviewer (name, role and inability to answer care-related questions)
- Explain what SDM is and the main aim of the research project (focus on what SDM is vs isn't, current problem and relevance/importance to community)
- Reminder of anonymity, confidentiality and that interview can be stopped at any time.
- *Take questions*
- Reminder of recording and check participant is happy
- *Switch on recorder*

Explore context of SDM experience

Having just explained a bit more about what the research aims to do and what good shared decision making looks like, I am interested to hear from you...

- What is your experience with decision making for any healthcare treatment?
- How much involvement in decisions about surgical treatment would you prefer?
- Do you feel that, for whatever reason, you felt that you were/would be treated unequal to others in terms of making decision?

Exploring intervention components

- What do you think about the hospital using this survey to record people's experiences of how involved they felt in the surgical decision making?
- How do you feel about personal survey responses being passed on to the clinical team and you/the patient not being anonymous?
- What do you think needs to happen next to make sure you/ patients are given a voice and SDM is improved?
- How well do you think this process would work for everyone?

For peer review only
